# Unraveling the mysteries of the gut-kidney axis: the protective role of traditional Chinese medicine in chronic kidney disease

**DOI:** 10.3389/fmicb.2025.1642377

**Published:** 2025-10-21

**Authors:** Jiahui Li, Lijia Diao, Menglin Li, Fugang Huang, Ke Sun

**Affiliations:** ^1^Haiyan Hospital of Traditional Chinese Medicine, Haiyan, China; ^2^The First School of Clinical Medicine, Zhejiang Chinese Medical University, Hangzhou, China; ^3^Jinhua Fifth Hospital, Jinhua, China; ^4^The Third School of Clinical Medicine, Zhejiang Chinese Medical University, Hangzhou, Zhejiang, China

**Keywords:** chronic kidney disease, traditional Chinese medicine, gut-kidney axis, therapeutic strategy, gut microbiota, uremic toxins, prebiotic and probiotic foods

## Abstract

Chronic kidney disease (CKD) constitutes a globally progressive nephropathy orchestrating inexorable deterioration of renal architecture. The paradigmatic gut-kidney axis unveils sophisticated bidirectional interplay between enteric microbiome and renal homeostatic equilibrium. Dysbiotic perturbations catalyze aberrant accumulation of gut-derived uremic metabolites, attenuate intestinal epithelial fortification, and propagate subclinical inflammatory cascades, synergistically precipitating CKD trajectory acceleration. Contemporary therapeutic arsenals targeting this axis encompass probiotic reconstitution, prebiotic orchestration, synbiotic hybridization, precision nutritional calibration, and enteral sequestrants including AST-120. Traditional Chinese Medicine (TCM) paradigms deploy multifaceted strategies through meticulous microbiota choreography, mucosal barrier reinforcement, and renal fibrogenesis attenuation. Distinguished phytotherapeutics including Rhubarb (Rhei Radix et Rhizoma), *Salvia miltiorrhiza*, and Poria cocos, synergized with bioactive constituents curcumin and punicalagin, orchestrate nephroprotective virtuosity via intricate microbiome-metabolome networks. Sophisticated polyherbal architectures, exemplified by YQHG and YSHS, harmoniously fortify intestinal impermeability while nullifying uremic translocation. These revelations substantiate the transformative potential of integrative paradigms amalgamating TCM-based phytomedicine with microbiota-directed precision therapeutics for CKD stewardship.

## 1 Introduction

Chronic Kidney Disease (CKD) constitutes a multifaceted clinical entity characterized by the inexorable deterioration of renal architecture and physiological function, representing one of the most formidable challenges in contemporary nephrology (D’Arrigo et al., 2025; Tsinari et al., 2025). This complex pathological syndrome may manifest as a sequela to diverse primary renal pathologies, including diabetic nephropathy (DN) and IgA nephropathy (IgAN), with its relentless progression orchestrated through cascading mechanisms of irreversible nephron depletion, progressive glomerular sclerosis, and extensive tubulointerstitial fibrosis, inevitably culminating in end-stage renal disease (ESRD) (Huang et al., 2025; Kitamura et al., 2025; Tang et al., 2025). The clinical phenotype of CKD is predominantly characterized by the triad of persistent proteinuria, hematuria, and progressive decline in estimated glomerular filtration rate (eGFR). Contemporary epidemiological surveillance reveals an alarming trajectory of escalating global CKD prevalence, currently imposing its burden upon more than 10% of the worldwide adult demographic (Selby and Taal, 2024). Particularly noteworthy is CKD’s propensity to transcend the boundaries of isolated renal dysfunction, precipitating a constellation of cardiovascular complications and metabolic derangements that collectively contribute to substantially elevated all-cause mortality, thereby underscoring the imperative for innovative therapeutic paradigms.

Contemporary therapeutic modalities remain predominantly anchored to renal replacement strategies, encompassing dialysis and renal transplantation; however, the inherent limitations of these interventions—including their invasive nature, prohibitive healthcare expenditures, and fundamental inability to reverse underlying pathophysiological processes—severely constrain their therapeutic utility and accessibility (Wieringa et al., 2025). Within this therapeutic lacuna, personalized treatment strategies rooted in traditional medicinal systems, particularly the sophisticated theoretical framework of Traditional Chinese Medicine (TCM), have emerged as compelling alternatives. TCM’s therapeutic philosophy, predicated upon multi-target synergistic modulation and holistic restoration of physiological equilibrium, demonstrates considerable promise in attenuating CKD progression and ameliorating symptomatic burden, thereby providing a robust theoretical foundation for integrative Chinese-Western medical approaches.

The paradigmatic evolution of CKD pathogenesis research has witnessed a transformative shift from reductionist single-organ conceptualization toward sophisticated multi-systemic network modeling, epitomized by the revolutionary “gut-kidney axis” hypothesis (Tsuji et al., 2024; Kemp et al., 2025). This groundbreaking theoretical construct elucidates the intricate bidirectional regulatory networks governing intestinal microecological dynamics and renal physiological homeostasis. Within the pathological milieu of uremia-associated CKD, the intestinal microbiome undergoes profound compositional dysregulation, manifesting as precipitous depletion of beneficial microbial taxa—particularly short-chain fatty acid (SCFA)-producing genera including *Faecalibacterium prausnitzii*, Roseburia, Bifidobacterium, and Lactobacillus—concomitant with the aberrant proliferation of opportunistic pathogenic species such as *Escherichia coli*, Shigella, and Enterococcus (Lyu et al., 2023; Atzeni et al., 2024; Wakino et al., 2025). This dysbiotic transformation precipitates compromised SCFA biosynthetic capacity, disruption of intestinal epithelial barrier integrity, and the pathological phenomenon of intestinal hyperpermeability or “leaky gut syndrome.” The consequent translocation of gut-derived pathogen-associated molecular patterns (PAMPs), exemplified by lipopolysaccharides, orchestrates chronic low-grade inflammation through Toll-like receptor (TLR) activation and cascading pro-inflammatory cytokine release, thereby accelerating epithelial-mesenchymal transition within renal tubular epithelium and promoting progressive interstitial fibrogenesis (Rukavina Mikusic et al., 2020; Stasi et al., 2025). Moreover, the accumulation of deleterious microbial metabolites—including indoxyl sulfate (IS), p-cresyl sulfate (PCS), and trimethylamine N-oxide (TMAO)—exerts direct nephrotoxic effects through sophisticated mechanisms involving epigenetic dysregulation and oxidative stress amplification, establishing a self-perpetuating pathological feedback circuit of aberrant “gut-kidney crosstalk” (Alvarenga et al., 2025). Within the context of ESRD and maintenance dialysis, microbial community architecture exhibits additional layers of complexity, characterized by disease-specific, stage-dependent, and etiology-related compositional signatures. This revolutionary theoretical framework not only provides unprecedented insights into the systemic pathophysiology of CKD but also establishes a conceptual bridge between the holistic therapeutic principles of TCM and precision microbiome-targeted interventional strategies.

## 2 The gut-kidney axis: orchestrating the pathogenesis of CKD

### 2.1 Disrupted gut microbiota: a catalyst for CKD onset

The perturbation of gut microbiota homeostasis has emerged as a pivotal orchestrator in the pathogenesis of CKD, manifesting through temporally-orchestrated microbial compositional metamorphoses characterized by the quintessential depletion of SCFA-producing commensals and the concomitant proliferation of opportunistic pathogens (Evenepoel et al., 2023; Krukowski et al., 2023; Zheng et al., 2023; Stepanova, 2024; Zhao B. et al., 2025).

Foundational investigations in animal paradigms have elucidated that renal deterioration in membranous nephropathy rodents correlates with the precipitous decline of *Lactobacillus* and *Bifidobacterium* populations (Miao et al., 2024b). The 5/6 nephrectomy murine archetype demonstrated amplified colonization by *Allobaculum*, *Bifidobacterium*, and *Turicibacter*, juxtaposed with the attenuation of *Lactobacillus* and *Rikenellaceae* constituents (Chen F. et al., 2025). The adenine-induced paradigm exhibited taxonomically-selective enrichment of genera encompassing *Dorea*, *Escherichia*, *Clostridium*, and *Ruminococcus* (Li et al., 2025). Nevertheless, the inherent physiological divergence between rodents—functioning as cecal fermenters with coprophagic proclivities—and humans, who rely predominantly on colonic fermentation, presents formidable translational impediments that may circumscribe the clinical applicability of these experimental findings (Jensen et al., 2023). From an anatomical and physiological perspective, the rodent cecum presents itself as a formidable digestive powerhouse, commanding substantial territorial dominance within the abdominal cavity. In the murine paradigm, this remarkable organ claims an impressive 30%–40% of the entire gastrointestinal real estate, orchestrating vigorous peristaltic symphonies (Bolsega et al., 2023). Despite the ephemeral nature of luminal transit, this biological reactor sustains extraordinarily dynamic fermentative processes within its capacious chambers. The meticulously maintained acidic milieu creates an optimal sanctuary for specialized fibrolytic consortia, notably fostering the proliferation of *Akkermansia muciniphila* and allied mucin-degrading virtuosos that flourish in remarkable abundance. The intricate labyrinthine architecture of the cecal mucosa exponentially amplifies the colonization landscape for microbial inhabitants, while the delicately thin cecal epithelium facilitates the expeditious translocation of fermentation-derived SCFAs into systemic circulation. In striking contradistinction, the human colonic architecture embodies a more contemplative digestive philosophy, characterized by leisurely luminal transit and progressively constricting diameters that sculpt the distinctive haustral topography. This anatomical masterpiece orchestrates prolonged substrate retention, enabling the methodical and comprehensive deconstruction of complex carbohydrate matrices. The human colon manifests an elegant pH gradient cascade, thereby creating distinct ecological niches that strategically compartmentalize butyrate-producing populations proximally while concentrating ammonia-generating microorganisms distally (Cheng and Zhou, 2024; Xie Z. et al., 2024). The metabolomic fingerprints of these two biological systems reveal fascinatingly divergent signatures. Rodent fermentation chambers predominantly synthesize acetate-rich SCFA portfolios, with propionate occupying secondary prominence and butyrate assuming a more modest role—a metabolic choreography that epitomizes rapid high-fiber processing capabilities. Conversely, the human colonic fermentome exhibits a more sophisticated equilibrium, demonstrating reduced acetate predominance while elevating butyrate to greater prominence. This harmonious SCFA architecture reflects the complexity of omnivorous dietary adaptations and the intricate ecological networks governing human intestinal ecosystems, with butyrate emerging as an indispensable guardian of colonic epithelial barrier integrity through compelling clinical evidence. The secondary metabolite landscapes further illuminate these interspecies distinctions. Rodent bile acid biotransformation follows relatively streamlined pathways, predominantly yielding deoxycholic and lithocholic acid derivatives, whereas the human colonic microbiome orchestrates a more elaborate repertoire of secondary bile acid synthesis, encompassing ursodeoxycholic acid and numerous sophisticated metabolic derivatives. Similarly, tryptophan metabolic networks in human intestinal ecosystems generate remarkably diverse indole and derivative spectra, showcasing enhanced biochemical versatility. Despite these profound distinctions, both systems converge upon fundamental functional paradigms, particularly in energy metabolism orchestration, where both rodent and human microbiomes masterfully transform recalcitrant carbohydrates into bioavailable SCFAs, delivering vital energetic substrates to their respective hosts. Butyrate production exemplifies this functional convergence, serving as a pivotal homeostatic regulator that maintains intestinal equilibrium through the elegant induction of regulatory T-cell differentiation cascades in both species (Zhu et al., 2021), while propionate simultaneously modulates insulin sensitivity and lipid metabolism across phylogenetic boundaries (Jiao et al., 2021). The translational paradigm from rodent models to human clinical applications, while foundational to mechanistic understanding and therapeutic development, confronts formidable challenges rooted in these anatomical and physiological divergences. Probiotic strains demonstrating remarkable efficacy in murine systems may encounter colonization barriers or fail to replicate beneficial effects within the distinctive human intestinal landscape. Consequently, the scientific community must embrace increasingly sophisticated experimental architectures: deploying humanized microbiota mouse models to authentically recapitulate human intestinal ecosystems, implementing advanced *in vitro* colonic simulation platforms that faithfully mirror human fermentative environments, and pioneering novel biomarker discovery initiatives to enhance predictive accuracy of translational outcomes. Through these refined methodological innovations, researchers can architect more precise translational strategies, dramatically elevating the success trajectory from fundamental discovery to clinical implementation.

Clinical investigations have unveiled distinctive microbial trajectories in CKD cohorts: the inaugural disease phases are marked by profound diminution of SCFA-synthesizing taxa (encompassing *Prevotellaceae*, *Enterococcus*, and *Lactobacillus*), with the magnitude of this depletion demonstrating positive correlation with eGFR deterioration (Atzeni et al., 2024). Simultaneously, pathogenic bacterial assemblages (including *Enterobacteriaceae*, *E. coli*, and *Clostridioides*) undergo exponential expansion, perpetuating nephrotoxicity through the biosynthesis of uremic toxins including ammonia, indole, and para-cresol (Gryp et al., 2020b; Ahmed and Al-Massri, 2023). Particularly noteworthy is the elevation of *Bacteroidaceae* abundance, which demonstrates direct causality with uric acid/urea metabolic aberrations (Gryp et al., 2020b). As renal functionality undergoes progressive deterioration, anti-inflammatory microbial species such as *Roseburia* and *Faecalibacterium prausnitzii* experience sequential depletion (Yang C.-Y. et al., 2021; Cabała et al., 2024), while toxigenic *Clostridium* species undergo further amplification (Yang C.-Y. et al., 2021). Furthermore, the taxonomic lineage *Bacilli-Lactobacillales-Lactobacillaceae-Lactobacillus-Lactobacillus johnsonii* demonstrates positive correlation with CKD progression in clinical populations, with its abundance correlating with serum creatinine concentrations, and therapeutic supplementation of this strain conferring nephroprotective benefits (Miao et al., 2024a). This microbial dysregulation additionally predisposes to nephrolithiasis through oxalate metabolism perturbations, manifesting as augmented urinary oxalate excretion consequent to *Bifidobacterium* depletion and *Bacteroides* dominance (Cabała et al., 2024).

However, given the etiological heterogeneity encompassing primary and secondary CKD manifestations, the resultant gut microbiota perturbations exhibit pathophysiologically-distinct signatures (Li J. et al., 2024). In primary CKD complicated by nephrotic syndrome, patients afflicted with idiopathic membranous nephropathy (IMN) demonstrate pronounced enrichment of gram-negative pathogenic constituents including *Shigella* spp.*, Streptococcus* spp., *Enterobacter* spp., *Enterococcus* spp., and *Escherichia coli*, while experiencing marked attenuation of beneficial taxa such as *Lachnospira*, *Lachnospiraceae*, and *Veillonella*, accompanied by compromised SCFA biosynthesis, attenuated immunomodulatory capacity, and heightened inflammatory cascades (Zhang J. et al., 2020; Motavalli et al., 2021; Doré et al., 2022). Minimal change disease (MCD) patients manifest diminished microbial biodiversity with depleted butyrate-producing genera including *Faecalibacterium* and *Prevotella*, contrasted by pathogenic bacterial amplification such as *Escherichia-Shigella* (Zhang Y. et al., 2022). Focal segmental glomerulosclerosis (FSGS) paradigms demonstrate enhanced *Bifidobacterium*, *Collinsella*, and *Candida* colonization, while experiencing significant depletion of beneficial taxa including *Granulicatella, Christensenella, Turicibacter*, and *Rikenella*, culminating in elevated deleterious metabolite concentrations, compromised intestinal barrier integrity, endotoxemia, and sustained mTORC1 signaling pathway activation that perpetuates glomerular sclerosis and fibrogenesis (Parker et al., 2020; Nakano et al., 2021; Shi et al., 2023). Mesangial proliferative glomerulonephritis (MsPGN) patients exhibit augmented *Bradyrhizobium* and *Hyphomicrobium* populations, concomitant with diminished *Ruminococcaceae*, *Alistipes*, and *Lachnospira* representation (He et al., 2021).

Predominant secondary CKD etiologies encompass DN and lupus nephritis (LN). DN patients manifest attenuated gut microbial biodiversity, with pronounced Actinobacteria elevation at the phylogenetic level, accompanied by decreased *Alphaproteobacteria* and *Clostridia* populations. At the genus level, *Christensenella*, *Clostridium-XIVa*, and *Eisenbergiella* demonstrate enhanced abundance, with *Eisenbergiella* exhibiting positive correlation with glomerular sclerosis and basement membrane thickening (Lu et al., 2023). Gut microbial perturbations in DN demonstrate 63% metabolic association (Zhang B. et al., 2022; Zhao et al., 2024), characterized by diminished SCFA-producing bacteria (*Butyricicoccus*, *Faecalibacterium*, *Lachnospira*, *Roseburia*, *Ruminococcus*, *Coprococcus*, *Eubacterium*, and *Clostridium leptum*) and pronounced enrichment of pathogenic taxa *Hungatella*, *Bilophila*, and *Escherichia*, compromising serum and fecal SCFA concentrations, with this decline demonstrating positive correlation with renal dysfunction (Zhong et al., 2021). LN patients exhibit gut microbial dysregulation characterized by diminished Firmicutes/Bacteroidetes ratios, accompanied by enhanced *Proteobacteria*, *Streptococcus*, *Lactobacillus*, *Ruminococcus gnavus*, and *Lactobacillus reuteri* populations. *R. gnavus* abundance demonstrates nephrotrophic pathological predisposition, correlating positively with systemic inflammatory cascades, dysregulated inflammatory immune cell differentiation patterns, and autoantibody concentrations, while multi-strain *Lactobacillus* therapeutic intervention facilitates intestinal barrier restoration in LN murine models (Nepal and Gazeley, 2023; Wang A. et al., 2023; Hosseini et al., 2024). Additionally, IgA nephropathy, constituting a primary etiology with CKD progression potential, demonstrates elevated *Escherichia coli* abundance in affected patients relative to healthy controls, while altered *Enterococcaceae*, *Moraxella*, and *Acinetobacter* populations associate with renal dysfunction (Li X.-J. et al., 2024). IgA vasculitis with nephritis (IgAV-N) similarly progresses to CKD, exhibiting gut microbial dysbiosis characterized by enhanced potentially pathogenic bacteria including *Shigella* and *Streptococcus*, juxtaposed with diminished beneficial taxa such as *Prevotella* (Tan et al., 2022).

CKD patients advancing to ESRD necessitate dialytic intervention for survival. Microbial dysregulation in dialysis populations presents modality-specific perturbations: comprehensive *Firmicutes* depletion at the phylum level, accompanied by enhanced *Proteobacteria* and *Fusobacteria* abundance; diminished *Clostridia* representation at the class level with augmented *Gammaproteobacteria*; reduced *Clostridiales* abundance at the order level with enhanced *Enterobacteriales*; attenuated abundance of *Lactobacillaceae*, *Lachnospiraceae*, *Ruminococcaceae*, and *Bifidobacteriaceae* at the family level, contrasted by amplified *Enterobacteriaceae*, *Pseudomonadaceae*, and *Enterococcaceae*; diminished *Faecalibacterium*, *Roseburia*, *Bifidobacterium*, and *Prevotella* representation at the genus level, with enhanced *Escherichia*, *Shigella*, *Pseudomonas*, and *Enterococcus*; and reduced *Faecalibacterium prausnitzii* abundance at the species level accompanied by enhanced *Escherichia coli* (Bao et al., 2022; Shivani et al., 2022; Li J. et al., 2023; Teixeira et al., 2023; Stepanova, 2024). Distinct dialytic modalities generate divergent manifestations: hemodialysis patients characteristically exhibit *Actinobacteria*, *Firmicutes*, and *γ-Proteobacteria* proliferation, accompanied by pronounced depletion of butyrate-synthesizing taxa (including *Faecalibacterium* and *Roseburia*) (Gao et al., 2024; Yasuno and Ito, 2024), while peritoneal dialysis patients demonstrate *Enterobacteriaceae* amplification and *Bifidobacterium/Lactobacillus* depletion (James et al., 2025). These perturbations not only compromise intestinal barrier integrity but also exacerbate microinflammatory cascades through lipopolysaccharide (LPS) translocation, establishing a pernicious “gut-kidney axis deterioration cycle.” Furthermore, gut microbial dysregulation constitutes a cardinal pathogenic determinant in CKD-associated cardiorenal syndrome (CRS), with microbial metabolites such as TMAO compromising cardiac and renal functionality, precipitating inflammatory cascades and fibrogenesis (Zhao B. et al., 2025).

In synthesis, CKD-associated microbial dysregulation manifests through a paradigmatic trajectory of “beneficial taxa depletion—pathogenic taxa hegemony,” propelling disease progression via multifaceted mechanisms encompassing uremic toxin biosynthesis, metabolic dysfunction, and immunological dysregulation. Comprehensive elucidation of gut microbiota pathophysiological contributions in CKD establishes the theoretical foundation for revolutionary therapeutic paradigms predicated upon microbiome modulation (Gryp et al., 2020b; Yang C.-Y. et al., 2021; Cabała et al., 2024).

### 2.2 Dysbiosis-induced metabolic disruption and escalation of inflammatory responses

CKD patients manifest profound microbial biodiversity attenuation, accompanied by pathological proliferation of urease-synthesizing bacteria and proteolytic bacterial consortia. This ecological perturbation orchestrates intestinal homeostatic collapse through multifarious pathophysiological cascades (Li X.-J. et al., 2024). Bacterial urease-mediated ammonia (NH₃) biosynthesis precipitates dramatic colonic pH alkalinization (transitioning from physiological mild acidification to pathological alkalinity), consequently triggering aberrant goblet cell hypersecretion, compromising colonic epithelial barrier architectural integrity, and culminating in catastrophic intestinal permeability augmentation (Rukavina Mikusic et al., 2020). These pathological metamorphoses facilitate the systematic translocation of protein-bound uremic toxins (encompassing IS and PCS) and bacterial-derived metabolites (including LPS) into systemic circulation via portal venous networks. Concomitantly, CKD patients exhibit profound downregulation of organic anion transporter OAT1/OAT3 expression within renal and hepatic parenchyma, accompanied by compromised efflux pump BCRP and MRP2/4 functionality, ultimately precipitating catastrophic systemic uremic toxin bioaccumulation (Masereeuw, 2022; Spicher et al., 2025; You et al., 2025). These accumulated nephrotoxic moieties demonstrate remarkable capacity to traverse formidable biological barriers including the blood–brain and blood-pancreatic barriers, orchestrating deleterious multi-organ system perturbations encompassing the central nervous system, pancreas, and skeletal musculature through sophisticated epigenetic modifications and pivotal signaling pathway dysregulation, ultimately catalyzing systemic endotoxemia characterized by relentless low-grade inflammatory cascades and profound immunological dysregulation, establishing a pernicious pathophysiological vortex that exponentially accelerates renal functional deterioration (Bossola and Picconi, 2024; Cabała et al., 2024; He et al., 2024; Xie H. et al., 2024; Chen M.-C. et al., 2025).

Urea, functioning as the quintessential uremic metabolite orchestrating CKD progression, undergoes profound metabolic pathway remodeling during renal functional decimation: as estimated glomerular filtration rate (eGFR) experiences precipitous decline, urea excretory mechanisms undergo dramatic transition from renal dominance to compensatory colonic clearance modalities (Tsuji et al., 2024). This pathological compensatory paradigm precipitates dose-dependent colonic urea concentration escalation, systematically inhibiting SCFA-producing bacterial proliferation while selectively promoting urease-producing pathogenic bacterial colonization supremacy, establishing a devastating “urea bioaccumulation–dysbiotic amplification” positive feedback vortex (Li Y. et al., 2023; Pyatchenkov et al., 2023). Urea and its cytotoxic degradation product ammonia orchestrate submucosal M1 macrophage polarization and catalyze pro-inflammatory mediator cascade release (González-Guerrero et al., 2017; Qian, 2017; Kim et al., 2025; Shentu et al., 2025); simultaneously, these metabolites systematically downregulate tight junction protein Claudin-1 and ZO-1 phosphorylation homeostasis, inducing epithelial cell junctional complex architectural disintegration and exponentially augmenting bacterial DNA and endotoxin translocation vulnerability (Wang and Gao, 2022; Kane et al., 2023). Remarkably, reactive oxygen species (ROS) generated through colonic urea metabolic processes demonstrate capacity to induce renal tubular epithelial cell mitochondrial functional catastrophe, exponentially accelerating tubulointerstitial fibrotic transformation and establishing a devastating “gut-derived urea–renal injury” pathophysiological cascade amplification phenomenon (Rukavina Mikusic et al., 2020; Pissas et al., 2024; Yang et al., 2025).

Intestinal microbial dysregulation additionally orchestrates protein-bound toxin biosynthetic amplification through aberrant aromatic amino acid metabolism (encompassing tryptophan, tyrosine, and phenylalanine), synthesizing IS, indole-3-acetic acid (IAA), PCS, and phenylacetylglutamine. Sophisticated clinical cohort investigations have definitively established that plasma concentrations of these nephrotoxic moieties demonstrate profound positive correlation with complication incidence trajectories and all-cause mortality paradigms in CKD populations (Yang C.-Y. et al., 2021; Cabała et al., 2024), with IS and PCS functioning as sophisticated biomarkers that exquisitely reflect renal functional decline kinetics, assess renal functionality and disease severity gradations, demonstrating exceptional concordance with CKD staging classifications (Corradi et al., 2024).

IS orchestrates endothelium-dependent vasodilation impairment through sophisticated AhR receptor binding mechanisms and nitric oxide synthase activity inhibition; its extraordinary albumin-binding affinity renders it virtually recalcitrant to conventional hemodialytic clearance modalities (Gryp et al., 2020a; Nguyen et al., 2022; Sánchez-Ospina et al., 2024). At the molecular architectural level, IS executes extraordinarily inflammatory signaling orchestration within renal parenchymal microenvironments through multifarious convergent pathophysiological cascades: it facilitates NF-κB nuclear translocation dynamics via toll-like receptor 4 (TLR4) activation paradigms in renal tubular epithelial cellular matrices (Pei et al., 2022), concurrently catalyzing NLRP3 inflammasome architectural assembly and subsequent IL-1β and IL-18 cytokine liberation cascades (Yamaguchi et al., 2022). Furthermore, IS orchestrates p38 MAPK and ERK1/2 phosphorylation signaling networks, culminating in amplified biosynthesis of pro-inflammatory cytokine arsenals encompassing TNF-*α*, IL-6, and MCP-1 (Ribeiro et al., 2023). This nephrotoxic moiety additionally activates aryl hydrocarbon receptor (AhR) signaling architectures within podocyte and mesangial cellular compartments, inducing CYP1A1 expression paradigms while exacerbating oxidative stress microenvironments and promoting TGF-β1 transcriptional upregulation, thereby exponentially accelerating glomerulosclerotic progression and tubulointerstitial fibrotic transformation trajectories (Iwashima et al., 2024; Nanto-Hara and Ohtsu, 2024).

p-Cresol (PCS), constituting a sophisticated tyrosine metabolism byproduct, systematically compromises renal functionality through oxidative stress amplification and inflammatory response orchestration, with its sulfate and glucuronide derivatives exhibiting extraordinary nephrotoxic potency and demonstrating relentless systemic bioaccumulation, exponentially exacerbating tissue injury (Sánchez-Ospina et al., 2024; Renaldi et al., 2025). PCS demonstrates extraordinary proficiency in activating renal inflammatory network architectures through complementary yet mechanistically distinct pathophysiological paradigms: it promotes ROS generation via NADPH oxidase activation cascades within renal tubular epithelial cellular matrices, simultaneously catalyzing JNK and p38 MAPK signaling pathway orchestration (Chang et al., 2020). PCS additionally activates NF-κB pathway architectures through IκB kinase (IKK) phosphorylation mechanisms, precipitating enhanced transcriptional activation of pro-inflammatory genetic programs encompassing COX-2, iNOS, and diverse chemokine repertoires (Kuang et al., 2022). This metabolite further disrupts mitochondrial bioenergetic homeostasis through complex III inhibition paradigms, precipitating amplified superoxide production cascades and subsequent activation of redox-sensitive transcription factor networks, ultimately culminating in enhanced expression of profibrotic mediator arsenals (Al Khamici et al., 2022; Mishra et al., 2024).

Sophisticated research paradigms have elucidated that plasma tryptophan concentrations demonstrate positive correlations with *Turicibacter*, *Clostridium IV*, *Pseudomonas*, and *Lactobacillales,* while exhibiting inverse correlations with *Blautia*, *Oscillibacter*, and *Intestinimonas*. These latter genera harbor sophisticated genetic arsenals encoding pivotal tryptophan metabolism enzymes (including tryptophan synthase K16187, indoleamine-2,3-dioxygenase IDO K00463, and tryptophan-2,3-dioxygenase TDO K00453) and their corresponding enzymatic machinery (EC:1.13.11.52 and EC:1.13.11.11), which systematically amplify tryptophan metabolite biosynthesis, thereby exponentially accelerating renal fibrotic progression trajectories. In 5/6 nephrectomy rodent paradigms, attenuated serum tryptophan concentrations and elevated plasma concentrations of tryptophan metabolites demonstrate positive correlations with intestinal *Clostridium IV* abundance while exhibiting inverse correlations with *Blautia*, *Enterorhabdus*, *Allobaculum*, *Clostridium sensu stricto*, and *Escherichia shigella* populations (Selby and Taal, 2024). Furthermore, tryptophan metabolic aberrations have been meticulously characterized in DN and membranous nephropathy paradigms (Gooding et al., 2019). In DN pathophysiology, amplified *Escherichia* abundance intensifies tryptophan metabolic cascades, generating nephrotoxic indole and its derivative IS, exponentially exacerbating renal functional deterioration (Li J. et al., 2024); in membranous nephropathy contexts, diminished *Lactobacillus johnsonii, L. reuteri, L. vaginalis, L. murinus*, and *Bifidobacterium animalis* populations demonstrate positive correlations with IAld, indole-3-pyruvate, and tryptamine mortality trajectories while exhibiting inverse correlations with elevated IAA and ILA concentrations (Miao et al., 2024b).

Additionally, TMAO (synthesized through microbial metabolism of dietary choline and carnitine precursors) demonstrates profound associations with atherosclerotic progression trajectories and cardiovascular risk amplification (Zhen et al., 2023). Within renal cellular microenvironmental, TMAO orchestrates extraordinarily inflammatory activation paradigms through multifarious complementary mechanistic networks: it amplifies scavenger receptor CD36 expression dynamics within macrophage populations infiltrating renal interstitial compartments, promoting foam cell morphological transformation and subsequent inflammatory mediator liberation cascades (Luo et al., 2024). TMAO additionally catalyzes NLRP3 inflammasome pathway activation within renal tubular epithelial cellular matrices through potassium efflux-dependent mechanistic paradigms, precipitating caspase-1 activation cascades and enhanced IL-1β processing machinery (Kapetanaki et al., 2021). Furthermore, TMAO promotes endoplasmic reticulum stress responses within podocyte cellular compartments via IRE1α and PERK pathway activation architectures, culminating in amplified production of inflammatory cytokine arsenals and accelerated apoptotic cell death trajectories (Pan et al., 2023). This metabolite concurrently activates vascular smooth muscle cell proliferative cascades through PDGF receptor signaling orchestration while promoting endothelial dysfunction via eNOS uncoupling mechanisms and enhanced superoxide production paradigms (Brunt et al., 2020). In DN pathophysiology, intestinal *Hungatella* populations and their progenitor bacterium *Clostridium hathewayi* orchestrate TMAO biosynthetic amplification, catalyzing inflammatory cascade activation and augmenting oxidative stress paradigms alongside renal fibrotic transformation (Li J. et al., 2024); altered *Streptococcus* abundance demonstrates positive correlations with hypoalbuminemic states and hematuria manifestations in IgAV-N patients, while *Escherichia–Shigella* populations synthesize LPS that achieves systemic circulation, activating sophisticated immune response cascades and directly orchestrating glomerular endothelial and renal tubular epithelial cell injury (Li J. et al., 2024). In advanced CKD necessitating dialytic intervention, the metabolic landscape undergoes catastrophic transformation, with previously delineated trends in SCFA, TMAO, IS, and p-cresol concentrations becoming exponentially severe (Bao et al., 2022; Li J. et al., 2023). Illustratively, diminished beneficial bacterial populations including *Faecalibacterium*, *Roseburia*, and *Bifidobacterium*-induced SCFA biosynthetic deficiency precipitates systemic inflammatory cascade activation and cardiovascular risk amplification in PD patients (Jiang et al., 2021; Li J. et al., 2023). Conversely, pathological expansion of potentially pathogenic bacterial populations including *Escherichia*, *Shigella*, and *Enterococcus* may exponentially augment peritonitis susceptibility and infection vulnerability in PD populations (Zhou et al., 2022). Phenylacetylglutamine (derived through phenylalanine metabolic pathways) demonstrates profound elevation in advanced CKD and dialytic patients, augmenting arrhythmic vulnerability through cardiomyocyte calcium homeostatic disruption (Wang and Gao, 2022; Kane et al., 2023).

The pathophysiological manifestations of these gut-derived nephrotoxic moieties exhibit sophisticated multi-organ network characteristics: within intestinal microenvironments, toxin bioaccumulation exacerbates barrier dysfunction through tight junction protein complex architectural disruption and intestinal epithelial cell apoptotic cascade induction; systemically, these toxins orchestrate chronic inflammatory microenvironmental establishment through monocyte surface TLR activation and NF-κB signaling cascade initiation; within renal parenchymal architecture, toxins exponentially accelerate tubular atrophic transformation and interstitial fibrotic progression through mitochondrial oxidative phosphorylation uncoupling mechanisms and endoplasmic reticulum stress pathway activation (Chermiti et al., 2024; Frąk et al., 2024; Li X.-J. et al., 2024; Peris-Fernández et al., 2024a, 2024b). Furthermore, the prevalent delayed colonic transit phenomenon in CKD populations systematically exacerbates microbial dysbiotic states and metabolic perturbations by prolonging toxin-mucosa interface duration (Talamantes et al., 2024). Contemporary mechanistic investigations have revealed that IS orchestrates sophisticated disruption of mitochondrial fission/fusion architectural dynamics, systematically impedes physiological mitochondrial biogenetic processes, interferes with mitochondrial electron transport chain functionality, while compromising mitochondrial quality control homeostatic mechanisms, fundamentally remodeling gut microbiota metabolic phenotypic expressions, illuminating the pivotal significance of “microbiota–mitochondria cross-talk” paradigms in uremic toxin bioaccumulation phenomena (Lu et al., 2025; Rumanli et al., 2025).

**Figure 1:**
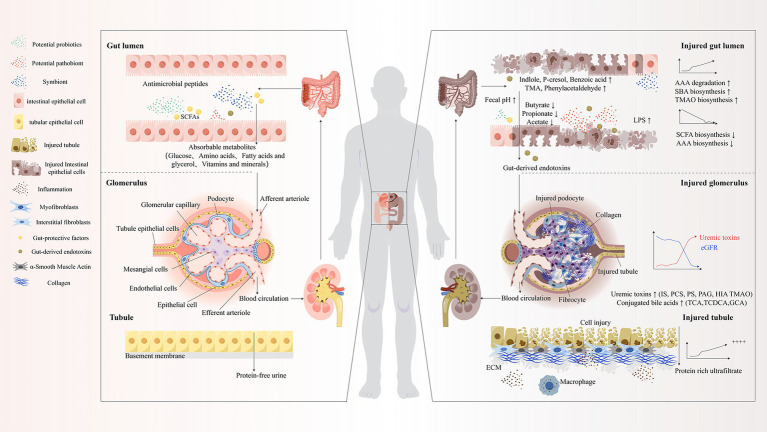
“Gut-Kidney Axis” theory. SCFAs, Short-Chain Fatty Acids; TMA, Trimethylamine; AAA, Aromatic Amino Acids; SBA, Secondary Bile Acids; TMAO, Trimethylamine N-oxide; eGFR, Estimated Glomerular Filtration Rate; IS, Indoxyl Sulfate; PCS/PS, p-Cresyl Sulfate; PAG, Phenylacetic Acid; HIA, Hippuric Acid; TCA, Tricarboxylic Acid; TCDCA, Taurochenodeoxycholic Acid; GCA, Glycocholic Acid; ECM, Extracellular Matrix; LPS, Lipopolysaccharide.

This “intestinal microbial dysregulation–uremic toxin bioaccumulation–multi-organ injury cascade” constitutes the cardinal pathophysiological circuitry orchestrating CKD progression trajectories (Figure 1). Comprehensive elucidation of the sophisticated molecular mechanisms governing pivotal nodes within this intricate system (encompassing urease activity regulatory paradigms, tight junction protein post-translational modification networks, and toxin receptor signal transduction cascades) will establish the theoretical foundation for developing revolutionary multi-target therapeutic strategies specifically targeting the sophisticated “gut–kidney axis” pathophysiological network.

### 2.3 Technological insights into gut-kidney crosstalk

Contemporary breakthroughs in organ-on-a-chip biotechnology have unveiled the exquisitely orchestrated bidirectional crosstalk networks between gastrointestinal and renal physiological systems (Wang et al., 2024). Pioneering experimental paradigms utilizing gut-on-a-chip microfluidic platforms have demonstrated that exposure to intestinal barrier-compromising agents, encompassing bacterial lipopolysaccharides and pro-inflammatory cytokine cascades, precipitates quantifiable augmentation of epithelial barrier permeability dynamics. Concurrent investigations employing cutting-edge kidney-on-a-chip architectures have revealed that renal parenchymal tissues demonstrate remarkable adaptive responsiveness to gut-derived metabolic signaling through modulation of transport protein expression repertoires, representing an elegant adaptive mechanism meticulously calibrated to preserve systemic metabolic equilibrium (Jochems et al., 2019; Langerak et al., 2020).

This revolutionary technological paradigm has catalyzed unprecedented revelations into the intricate molecular choreography governing inter-organ communication networks, particularly illuminating the cardinal significance of microbiota-derived metabolites in orchestrating both physiological homeostatic maintenance and CKD pathogenesis trajectories. The seamless integration of these sophisticated microfluidic organ modeling systems establishes an extraordinarily refined experimental framework for elucidating the mechanistic foundations underlying gut-kidney axis dysregulation phenomena, potentially catalyzing the emergence of precision-targeted therapeutic interventions specifically designed to combat chronic kidney disease pathophysiology.

## 3 Therapeutic strategies targeting the gut-kidney axis: current advances

Therapeutic paradigms predicated upon gut-kidney axis pathophysiology have undergone sophisticated evolution into comprehensive, multidimensional intervention architectures. This encompasses an arsenal of microbiota-modulatory agents (encompassing probiotics, prebiotics, and synbiotic formulations), precision dietary compositional strategies, physisorption-based therapeutic modalities (including advanced adsorption technologies), and sophisticated uremic toxin elimination protocols. Each therapeutic modality demonstrates distinctive mechanistic profiles and clinical applications, collectively establishing a holistic therapeutic framework for optimizing renal functionality through meticulously calibrated interventions.

### 3.1 Microbiota-modulating agents

#### 3.1.1 Probiotic orchestration of intestinal homeostasis

Probiotics constitute an elite consortium of beneficial bacterial assemblages endowed with extraordinary capacity to orchestrate comprehensive intestinal microecological architectural transformation through colonization dynamics and competitive exclusion of deleterious pathogenic entities, thereby catalyzing profound enhancement of intestinal metabolic functionality and conferring multidimensional health optimization benefits (Ghasemzadeh, 2024). Distinguished probiotic taxonomic lineages encompass *Lactobacillus*, *Bifidobacterium*, *Propionibacterium*, *Bacillus*, *Akkermansia muciniphila*, *yeast species*, and *Streptococcus thermophilus* (Yang et al., 2022). Among these microbial virtuosos, *Lactobacillus* and *Bifidobacterium* are universally acclaimed as the foundational architectural cornerstones for engineering efficacious probiotic therapeutic masterpieces (Huang and Chen, 2024).

*Lactobacillus* demonstrates exquisite functionality through sophisticated lactic acid biosynthetic orchestration, precipitating strategic intestinal pH acidification that systematically inhibits acid-susceptible pathogenic proliferation while cultivating extraordinarily favorable intestinal microbial ecosystems (Leska et al., 2022). In elegant counterpoint, *Bifidobacterium* operates through sophisticated uremic metabolite biotransformation cascades, systematically attenuating toxin bioaccumulation while catalyzing SCFA biosynthesis through intricate carbohydrate fermentation choreography, further suppressing deleterious bacterial expansion (Anjana and Tiwari, 2022; de Carvalho et al., 2024). The exquisite synergistic interplay between *Lactobacillus* and *Bifidobacterium* transcends mere intestinal acid–base equilibrium maintenance, orchestrating comprehensive systemic health optimization through mechanisms encompassing antimicrobial compound biosynthesis, competitive nutritional and binding site monopolization, immune regulatory cellular activation cascades, and essential enzymatic and vitamin synthetic pathways (Tian et al., 2022).

The extraordinary therapeutic potential of probiotic interventions in end-stage CKD management has been meticulously substantiated through comprehensive research paradigms, demonstrating profound positive impacts on clinical trajectories (Soleimani et al., 2017; Yu et al., 2022; Huang and Chen, 2024; Ribeiro et al., 2024). Exemplifying this therapeutic excellence, clinical investigations in CKD stage 3–4 populations revealed that daily administration of meticulously engineered capsules containing 9 billion CFU probiotic consortiums (encompassing *Lactobacillus acidophilus KB27, Bifidobacterium longum KB31*, and *Streptococcus thermophilus KB19*) achieved remarkable reductions in blood urea nitrogen (BUN), creatinine, and uric acid concentrations while dramatically enhancing quality of life parameters with exceptional safety profiles (Ranganathan et al., 2010; Rahman et al., 2024). Particularly noteworthy, *Streptococcus thermophilus*, distinguished by extraordinary urease enzymatic activity, assumes pivotal significance in urea biotransformation and metabolic optimization choreography; its synergistic collaboration with *Lactobacillus* and *Bifidobacterium* generates superior metabolic byproducts, orchestrates intestinal environmental amelioration, and amplifies overall probiotic combination therapeutic magnificence (Arioli et al., 2024; Huang et al., 2024). Furthermore, probiotics facilitate comprehensive gastrointestinal symptom resolution in CKD populations (Yu et al., 2022) and potentially mitigate renal colic vulnerability associated with nephrolithiasis (Wigner et al., 2022). For advanced CKD patients, particularly those requiring hemodialytic interventions, pioneering research has demonstrated that *Bifidobacterium* encapsulation in acid-resistant delivery systems maintains extraordinary viability within hostile gastric acidic environments, enabling triumphant intestinal colonization and subsequent dramatic reductions in serum homocysteine, IS, and triglyceride concentrations while orchestrating profound renal functional restoration (Juárez-Trujillo et al., 2024; Schlienger de Alba and Espinosa Andrews, 2024). Wang et al.’s groundbreaking investigations demonstrated that probiotic interventions ameliorate GFR deterioration trajectories while achieving profound reductions in serum pro-inflammatory cytokine cascades, encompassing TNF-*α*, IL-6, and IL-18, in ESRD patients receiving hemodialytic therapy (Wang et al., 2021). Probiotics additionally orchestrate intestinal microbiota regulation to dramatically reduce peritonitis incidence, preserve residual kidney function, modulate inflammatory cascades, optimize nutritional parameters, and enhance health-related quality of life in advanced CKD patients requiring dialytic interventions (Stepanova, 2024).

Nevertheless, probiotic applications encounter sophisticated challenges requiring strategic resolution. Certain elite bacterial strains demand extraordinarily stringent survival parameters, including precisely calibrated pH environments or specialized hypoxic conditions, imposing elevated requirements for probiotic formulation engineering excellence and stability maintenance (Wigner et al., 2022). Despite extensive research establishing remarkable therapeutic potential in CKD management, consensus regarding optimal dosing paradigms, strain combination architectures, and treatment duration protocols remains elusive, while individual patient responses to probiotic interventions exhibit significant variability, further complicating standardized treatment protocol development. With revolutionary advancement in formulation biotechnologies, particularly groundbreaking achievements in strain stability enhancement and bioactivity amplification, alongside personalized probiotic combination engineering for CKD populations, probiotics are destined to assume transformative leadership roles in future nephrology therapeutics.

#### 3.1.2 Prebiotic ecological niche engineering mastery

Prebiotics constitute an elite class of functional non-viable substrates endowed with extraordinary capacity to undergo selective fermentation orchestration by specific intestinal probiotic virtuosos (particularly *Bifidobacterium* and *Lactobacillus*), thereby achieving profound intestinal microecological architectural metamorphosis. Prebiotics demonstrate ubiquitous presence within natural botanical matrices and, functioning as precision nutritional intervention modalities, exhibit unparalleled advantages in CKD populations confronting dietary protein restriction challenges (Tsai et al., 2019). This compound classification encompasses extraordinary diversity, including long-chain polyunsaturated fatty acids, fructooligosaccharide complexes, extended-chain inulin architectures, and resistant starch formulations, each possessing distinctive biochemical characteristics and therapeutic signatures (Gibson et al., 2017).

Prebiotics orchestrate probiotic proliferation enhancement, systematically inhibiting uremic toxin biosynthesis within colonic environments while dramatically amplifying SCFA generation cascades, thereby establishing intricate synergistic mechanisms specifically engineered to combat CKD-associated nephropathic processes (Tsai et al., 2019). Clinical investigations have demonstrated that meticulously engineered fructooligosaccharide-inulin composite prebiotic formulations achieve remarkable reductions in PCS biosynthetic rates and serum concentrations in advanced CKD populations (Mitrović et al., 2024; Yin et al., 2024). However, consequent to extraordinary compositional heterogeneity and sophisticated physiological interaction networks inherent to prebiotic architectures, definitive daily intake dosing paradigms specifically applicable to CKD populations remain unestablished. Clinical efficacy demonstrates profound influence from multifarious parameters, encompassing average polymerization degrees, dosing regimen protocols, treatment duration paradigms, and individual patient CKD disease progression stages, with these variables collectively orchestrating prebiotic intervention therapeutic performance trajectories.

#### 3.1.3 Synbiotic symphonic therapeutic orchestration

Synbiotics represent the absolute pinnacle of renal disease microecological intervention sophistication, constructing extraordinary synergistic therapeutic ecosystems through precision molecular matching of elite probiotic populations with meticulously targeted prebiotic substrate architectures. This revolutionary strategic paradigm generates sophisticated biological “symphonic masterpieces” within intestinal microecological landscapes, achieving unprecedented multi-dimensional precision interventions across comprehensive CKD pathophysiological networks. Synbiotic therapeutic excellence manifests through dual-mechanism orchestration: selective amplification of glycolytic beneficial bacterial populations while systematically suppressing proteolytic pathogenic bacterial consortiums characterized by potent protease enzymatic activity, thereby fundamentally reconstructing metabolic architectural landscapes within intestinal microenvironments, dramatically reducing uremic toxin burdens, restoring sophisticated immune homeostatic equilibrium, suppressing inflammatory cascade networks, and orchestrating profound renal functional restoration (Kieffer et al., 2016; Plaza-Díaz et al., 2017; Liu et al., 2024).

Synbiotic therapeutic magnificence has been comprehensively validated through clinical research paradigms. Illustratively, meticulously engineered synbiotic formulations encompassing *Lactobacillus*, *Bifidobacterium breve*, and galactooligosaccharide complexes achieve remarkable reductions in patient serum toxic metabolite concentrations, particularly demonstrating extraordinary PCS elimination efficacy (Mitrović et al., 2024; Anegkamol et al., 2025). Diverse synbiotic combination architectures exhibit unique therapeutic signature profiles, exemplified by sophisticated formulations containing *Lactobacillus*, *Bifidobacterium*, *Streptococcus*, and inulin complexes effectively reducing indole-producing bacterial abundance, thereby dramatically attenuating indole-class metabolites intimately associated with renal injury cascades (Yang C.-Y. et al., 2021).

Synbiotic therapeutic actions transcend conventional toxin elimination paradigms, demonstrating extraordinary multidimensional advantages at metabolic regulatory orchestration levels. They catalyze intestinal microbial biosynthesis of essential B-complex vitamin complexes (encompassing folate and niacin architectures), thereby elevating serum folate concentrations while dramatically reducing triglyceride and homocysteine levels—the latter constituting critical cardiovascular risk determinants in CKD populations. Additionally, synbiotics enhance calcium bioavailability through sophisticated intestinal pH regulatory orchestration, thereby strengthening calcium-phosphorus binding dynamics and ameliorating phosphorus metabolic disorders ubiquitous in CKD pathophysiology. Most remarkably, synbiotics orchestrate enhanced intestinal microbiota oxalate metabolic capacity, achieving profound reductions in urinary oxalate excretion, thereby assuming pivotal significance in nephrolithiasis prevention and associated renal injury mitigation (Rossi et al., 2015a). This comprehensive synergistic mechanism constellation establishes synbiotics as revolutionary therapeutic paradigms in comprehensive renal disease management architectures.

Contemporary network meta-analytical investigations have unveiled therapeutic spectrum differentiations among diverse microecological intervention modalities: prebiotics demonstrate maximal efficacy in uremic toxin elimination, achieving standardized mean differences of −1.24 for IS concentrations; synbiotics exhibit extraordinary advantages in microinflammatory regulatory orchestration, achieving remarkable 28.6% CRP concentration reductions; while probiotics demonstrate exceptional efficacy in gastrointestinal symptom amelioration, exhibiting relative risk ratios of 2.17 (Yu et al., 2022). Contemporary research trajectories are advancing toward two pioneering frontiers: individualized treatment strategy engineering predicated upon sophisticated microbiome precision stratification paradigms, and revolutionary nanotechnology utilization for optimizing active ingredient colonic-targeted delivery system architectures to enhance bioavailability and stability parameters. These groundbreaking advancement trajectories are destined to elevate “gut-kidney axis” interventions from adjunctive therapeutic modalities to cardinal pillars of comprehensive CKD treatment paradigms, inaugurating a transformative renaissance era in sophisticated nephrology therapeutic excellence.

### 3.2 Dietary composition: the molecular symphony of therapeutic nutrition

Dietary intervention emerges as the cardinal therapeutic paradigm within contemporary precision medicine, constituting the architectural foundation of CKD management. Through the meticulous orchestration of bespoke nutritional algorithms—particularly the synergistic convergence of low-protein dietary regimens (LPD) and dietary fiber modalities (DF)—clinicians achieve unprecedented manipulation of gut microbiome plasticity, catalyzing the biosynthesis of salutogenic metabolites (notably SCFAs) while simultaneously orchestrating the systematic suppression of deleterious proteolytic fermentation cascades. This sophisticated intervention matrix stabilizes pathophysiological trajectories, decelerates nephrosclerotic progression, and fundamentally reconstitutes prognostic landscapes in CKD populations (Serrano et al., 2022; Watanabe et al., 2022; Chang et al., 2023).

#### 3.2.1 The metabolic virtuosity of low-protein dietary architecture

The therapeutic magnificence of LPD paradigms resides in their exquisite choreography of nutritional homeostasis and metabolic refinement—achieved through precision-engineered protein restriction synergistically enhanced by essential amino acid (EAA) and ketoacid analogue supplementation. Within its biochemical nucleus, this intervention masterfully attenuates intestinal protein hydrolytic fermentation substrates, eloquently diminishing the genesis of nephrotoxic nitrogenous metabolites including ammonia and hydrogen sulfide, thereby dramatically alleviating renal metabolic burden (Watanabe et al., 2022; Wang et al., 2023b).

This sophisticated nutritional strategy operates through an intricate constellation of variables governing intestinal protein assimilation dynamics—encompassing quantitative intake parameters, protein conformational architectures (particularly thermodynamic denaturation profiles), phylogenetic origins (botanical versus zoological), and the complex matrix of concurrent dietary constituents. This multidimensional orchestration profoundly modulates small intestinal absorption kinetics and colonic transit dynamics, ultimately determining therapeutic magnitude for CKD populations (Di Iorio et al., 2019).

Compelling evidence demonstrates this dietary paradigm’s extraordinary capacity to recalibrate the delicate equilibrium between protein catabolism and amino acid salvage networks, facilitating adaptive responses to protein-restricted nutritional landscapes while preserving fundamental physiological integrity (Garibotto et al., 2018).

Within the microbial dimension, low-protein intervention precipitates a remarkable architectural transformation of intestinal microbial ecosystems in advanced CKD (stages 3B-4): initially, it orchestrates strategic depletion of pro-inflammatory *Proteobacteria* populations while concurrently amplifying anti-inflammatory *Actinobacteria* representation; subsequently, it catalyzes selective proliferation of butyrate-synthesizing bacterial consortiums (encompassing *Lachnospiraceae*, *Prevotellaceae*, *Bifidobacteriaceae*, *Faecalibacterium*, and *Roseburia*) alongside anti-inflammatory genera (notably *Blautia* and *Bacteroides*); finally, it systematically suppresses colonization of pathogenic microorganisms implicated in uremic toxin synthesis, including *Akkermansia*, *Streptococcus*, and *Escherichia* (Di Iorio et al., 2019). This microbial metamorphosis manifests clinically as simultaneous attenuation of circulating uremic toxins and D-lactic acid—with therapeutic efficacy exhibiting precise dose-dependency relative to dietary protein consumption (Di Iorio et al., 2019).

Pioneering investigations into amino acid pharmacological nutrition reveal the extraordinary therapeutic potential of sulfur-enriched amino acid regimens (featuring methionine and cysteine). These molecular entities execute post-translational modifications that fundamentally reconstitute the functional landscape of intestinal microbiota in experimental CKD paradigms. Their biochemical signature manifests as pronounced attenuation of tryptophanase enzymatic activity, effectively disrupting uremic toxin biosynthetic cascades and ultimately preserving host physiological integrity (Lobel et al., 2020).

Advanced pharmacological elucidation illuminates the metabolic elegance of ketoacid analogues, which—through their sophisticated transamination chemistry—sequester excess amino nitrogen reservoirs, channeling them toward *de novo* essential amino acid biosynthesis. This remarkable biochemical transformation not only diminishes nitrogenous waste streams and uremic toxin production but significantly decompresses renal metabolic burden. The metabolic reprogramming orchestrated by ketoacids transcends mere protein catabolism inhibition, extending to comprehensive optimization of protein metabolic networks and amino acid recycling efficiency in CKD patients, culminating in enhanced renal function preservation and disease trajectory modification (Ammar, 2022; Faerber et al., 2023; Chen Z. et al., 2024).

#### 3.2.2 The metabolic orchestration of dietary fiber therapeutics

Dietary Fiber (DF) crystallizes as the quintessential therapeutic cornerstone within the nutritional architecture for CKD patients—a therapeutic modality whose profound clinical significance transcends its humble molecular origins. At its structural essence, DF encompasses an elegant consortium of carbohydrate architectures that ingeniously circumvent human digestive enzymatic systems (Jensen et al., 2024), functioning as molecular sentinels that fortify gastrointestinal barrier integrity, orchestrate intricate microecological symphonies within the gut, and dramatically decompress renal metabolic pathways. Meticulously engineered high-fiber nutritional protocols achieve masterful equilibrium—delivering abundant fiber while deftly navigating the treacherous terrain of potassium and phosphorus accumulation, thus epitomizing precision in dietary therapeutics.

Through modulation of intestinal microbial constellations responsible for uremic toxin metabolism and renal metabolite transformation, DF reveals its extraordinary therapeutic potential within CKD’s clinical landscape. At the molecular frontier, DF artfully reprograms the nitrogen metabolism blueprint of gut flora: primarily, it catalyzes metabolic alchemy wherein intestinal microorganisms transmute urea and amino acids into vital anabolic building blocks, thereby refining the architectural and functional elegance of microbial ecosystems (Bergen, 2015); secondarily, it strategically curtails indigestible carbohydrate availability within colonic environments, imposing selective pressure against amino acid fermentation pathways, consequently diminishing nephrotoxic metabolite biosynthesis (De Vadder et al., 2014; Rossi et al., 2015b).

This harmonized biological choreography enables DF to recalibrate intestinal pH homeostasis, safeguard molecular integrity of tight junction proteins (notably ZO-1 and Occludin), and ultimately fortify barriers against pathological intestinal permeability (Yokoo et al., 2021; Skinner et al., 2025). Concurrently, DF employs sophisticated nitrogen chelation chemistry to dramatically reduce hepatorenal nitrogen burden (Mardinoglu et al., 2015; Yang H. L. et al., 2021), attenuating systemic inflammatory cascades and culminating in profound reductions of all-cause mortality among CKD populations (Chen Y. et al., 2024; Rispoli et al., 2025).

Resistant Starch (RS)—a distinguished member of the DF therapeutic pantheon—demonstrates extraordinary clinical virtuosity. Compelling evidence illuminates RS’s capacity to significantly diminish circulating IS and PCS concentrations in end-stage CKD patients undergoing hemodialysis (Jia et al., 2021). In preclinical investigations, RS intervention demonstrates remarkable restorative potential, rehabilitating renal function and intestinal barrier architecture in adenine-induced CKD models, while concurrently reducing tubulo-interstitial pathology scores and eliminating uremic toxins from circulatory systems (Kingra et al., 2022; Shamloo et al., 2022; Zhang Y. et al., 2024).

The specialized fermentable fiber compounds Guar Gum (GG) and Partially Hydrolyzed Guar Gum (PHGG) reveal multidimensional therapeutic virtuosity in experimental CKD paradigms: initially, they engineer strategic reductions in serum nitrogenous waste products (urea/creatinine); subsequently, they selectively silence pro-inflammatory cytokine expression (TNF-*α*, IL-1β, IL-6) and fibrogenic molecular pathways (TGF-β1, Col1a1); finally, they cultivate beneficial microbiotic transformations—enhancing Lactobacilli colonization and SCFA (acetate, propionate, butyrate) production—thereby orchestrating comprehensive recalibration of intestinal microbial ecology (Hung and Suzuki, 2018). This multifaceted mechanistic constellation illuminates DF’s position as an unparalleled therapeutic modality in sophisticated CKD management.

#### 3.2.3 Avant-garde nutritional intervention paradigms

Additional personalized dietary intervention strategies demonstrate remarkable potential in reconstituting intestinal flora architecture and metabolic homeostasis in CKD populations. Research illuminates that sodium propionate (SP)—a distinguished SCFA representative—when administered via encapsulated formulations to maintenance hemodialysis patients, orchestrates immune-metabolic equilibrium through multidimensional pathways: it significantly attenuates pro-inflammatory parameters including CRP, IL-1, and IL-17, while simultaneously upregulating anti-inflammatory mediators such as IL-10, concurrently reducing gut-derived uremic toxin production (IS and PCS), neutralizing oxidative stress markers including malondialdehyde, and rebalancing insulin resistance and iron metabolism perturbations (Marzocco et al., 2018).

The therapeutic constellation of natural dietary compounds reveals honey polyphenols as extraordinary microbiome architects—these bioactive molecules fundamentally reconstitute intestinal microbial communities, particularly enriching *Bifidobacteriales* and S24_7 bacterial populations, thereby amplifying SCFA production cascades. This microbial metamorphosis translates into quantifiable improvements in renal structural integrity and metabolic parameters within experimental CKD paradigms (Cao et al., 2024).

Perhaps most compelling is the vegetarian dietary paradigm—a comprehensive nutritional approach associated with dramatically reduced CKD prevalence and progression trajectories (Liu et al., 2019; Hao et al., 2024). Its multifaceted nephroprotective mechanism emerges through dual pathways: initially, the inherently minimal endogenous acid load characteristic of plant-predominant nutrition effectively counterbalances metabolic acidosis that typically accelerates renal functional deterioration in CKD (Chauveau et al., 2019; Carrero et al., 2020); subsequently, this dietary architecture dramatically attenuates circulating concentrations of nephrotoxic metabolites, including IS, PCS, and nitrogenous waste products such as serum urea (Kandouz et al., 2016; Cases et al., 2019).

While these personalized nutritional strategies illuminate a revolutionary therapeutic horizon in nephrology, critical challenges persist at the intersection of scientific understanding and clinical implementation. The mechanistic convergence between these diverse dietary interventions and prebiotic approaches necessitates more precise delineation of their unique therapeutic contributions. Furthermore, the translational pathway from compelling research findings to standardized clinical protocols remains incompletely navigated. The future research imperative lies in systematically validating these interventions through rigorous clinical trials, establishing evidence-based guidelines that stratify patients according to optimal intervention profiles, and exploring synergistic combinations with pharmacological approaches—ultimately elevating personalized nutritional medicine from a promising adjunct to the cornerstone of comprehensive CKD management.

### 3.3 Physisorption

AST-120, an ingeniously engineered oral activated carbon adsorbent, orchestrates the selective molecular capture of uremic toxin precursors (including indole and p-cresol) within the intestinal luminal microenvironment through physical adsorption mechanisms. This elegant intervention paradigm prevents transcellular absorption across intestinal epithelial barriers and subsequent hepatic sulfation cascades, culminating in dramatic attenuation of circulating indoxyl sulfate (IS) and p-cresyl sulfate (PCS) concentrations (Asai et al., 2019).

The therapeutic virtuosity of this extraordinary pharmaceutical compound transcends conventional renal boundaries, as illuminated by preclinical investigations that reveal a comprehensive constellation of multi-organ cytoprotective effects. Through its strategic molecular sequestration capabilities, AST-120 orchestrates a protective shield encompassing not merely renal parenchymal tissues, but extends its cytoprotective aegis to cardiac myocytes, cerebral neural networks, and skeletal muscle fiber architectures—collectively neutralizing the cytotoxic onslaught of uremic metabolites (Sato et al., 2017).

Expansive clinical investigations substantiate that AST-120 significantly decelerates eGFR deterioration through intricate mechanistic pathways involving systematic suppression of inflammatory cascades and oxidative stress networks (Cha et al., 2016; Chen et al., 2019). This nephroprotective efficacy emerges from the compound’s extraordinary capacity to disrupt the pathophysiological axis linking gut-derived toxin generation with systemic organ dysfunction.

While this therapeutic modality exemplifies an exceptional safety architecture—with adverse manifestations predominantly circumscribed to transient and benign gastrointestinal perturbations—the pursuit of optimized clinical outcomes reveals substantial interindividual heterogeneity in therapeutic responsiveness. This pharmacological variability illuminates the imperative for strategic therapeutic synergy with complementary interventions, particularly LPD regimens and precision-targeted pharmacological approaches, to achieve maximal therapeutic convergence across phenotypically diverse patient populations.

Through this orchestrated multimodal therapeutic convergence, AST-120 emerges as the quintessential cornerstone within the revolutionary paradigm of comprehensive uremic toxin neutralization—representing not merely an adjunctive intervention, but rather the architectural foundation upon which next-generation nephroprotective strategies are constructed.

### 3.4 Precision-orchestrated therapeutic architectures for uremic toxin neutralization

A constellation of pharmacological interventions orchestrates colonic uremic toxin metabolism through exquisitely distinct mechanistic pathways: the synergistic convergence of folic acid and methylcobalamin administration catalyzes homocysteine metabolic recalibration, systematically attenuating serum asymmetric dimethylarginine (ADMA) concentrations—a formidable mediator of endothelial dysfunction and cardiovascular pathophysiology (Angelini et al., 2021; Mohan et al., 2023); acarbose—the quintessential *α*-glucosidase inhibition virtuoso—precipitates a profound colonic metamorphosis by enriching the luminal microenvironment with undigested carbohydrate substrates, thereby amplifying SCFA biosynthetic cascades while simultaneously orchestrating the systematic suppression of bacterial deamination processes (Evenepoel et al., 2006); allopurinol, with its exquisite mechanistic precision, conducts a targeted biochemical silencing of xanthine oxidase enzymatic networks, fundamentally disrupting the pathophysiological cascade responsible for uric acid generation (Shuvo et al., 2025); meclofenamate emerges as a molecular disruptor, interfering with sulfotransferase-mediated sulfate conjugation mechanisms, thereby systematically reducing indolic toxin biosynthesis within intestinal compartments (Saigo et al., 2014); cilastatin orchestrates a strategic interruption of OAT1/3 functionality, constructing a protective molecular barrier that shields vital renal architectural elements from the cytotoxic assault of nephrotoxic pharmaceutical compounds (Huo et al., 2019).

These precision-engineered therapeutic modalities transcend the inherent limitations of conventional dialytic approaches in protein-bound toxin elimination, establishing a revolutionary multidimensional framework for toxin neutralization that surpasses the physical constraints of extracorporeal filtration methodologies. Through this orchestrated pharmacological symphony, clinicians achieve unprecedented control over uremic toxin burden, fundamentally redefining the therapeutic landscape of advanced CKD management.

### 3.5 Vanguard technologies and their revolutionary therapeutic potential

Within the pioneering realm of experimental therapeutics, fecal microbiota transplantation (FMT) crystallizes as a paradigmatic biologic intervention—orchestrating the systematic transfer of intricate microbial ecosystems from meticulously screened healthy donors to recipients harboring dysbiotic intestinal landscapes. This revolutionary approach to ecological restoration precipitates a comprehensive architectural reconfiguration of the intestinal microbiome, demonstrating extraordinary capacity to systematically diminish uremic toxin biosynthesis through precision-guided microbial community engineering (Caggiano et al., 2023). This living pharmaceutical paradigm harnesses the metabolic virtuosity of indigenous microbial consortiums to address the fundamental dysbiotic pathophysiology underlying uremic toxin generation networks. Nevertheless, the translational trajectory of FMT within CKD therapeutic paradigms confronts extraordinarily formidable multidimensional impediments that attenuate its immediate clinical implementation prospects. The exquisite sophistication of donor-recipient microbiome compatibility necessitates revolutionary algorithmic matching architectures that remain fundamentally underdeveloped, while the conspicuous absence of comprehensive longitudinal safety databases introduces profound uncertainty regarding potential iatrogenic consequences, encompassing the catastrophic risk of transmitting multidrug-resistant pathogenic organisms or occult oncogenic microbial constituents. Furthermore, the standardization of microbial preparation protocols presents unprecedented technical complexities, as the viability and therapeutic efficacy of transplanted microorganisms demonstrate extraordinary sensitivity to processing methodologies, cryopreservation technologies, and delivery system architectures. Bioethical considerations encompass intricate informed consent paradigms when implementing living microbial therapeutics of indeterminate compositional profiles, while regulatory frameworks encounter profound challenges accommodating this revolutionary therapeutic paradigm that transcends conventional pharmacological categorization schemas.

Organ-on-chip technology emerges as the quintessential masterpiece of biomimetic engineering—recreating the dynamic physiological interplay between intestinal and renal tissue architectures within exquisitely controlled microfluidic environments. These ingeniously engineered microphysiological systems have illuminated previously invisible pathways of organ crosstalk, revealing with unprecedented molecular clarity how intestinal barrier compromise initiates catastrophic signaling cascades across the gut-kidney physiological axis. Through these *in vitro* architectural platforms, researchers have achieved real-time visualization of how disrupted intestinal epithelial barriers facilitate the systematic translocation of inflammatory orchestrators (TNF-α, IL-6) and nephrotoxic mediators (IS) into systemic circulation, subsequently triggering progressive renal structural deterioration and functional collapse (Guo et al., 2023; Mou et al., 2024). However, the technical sophistication and prohibitive manufacturing expenditures associated with these microfluidic platforms severely circumscribe their accessibility and scalability for widespread research deployment across diverse institutional environments. Moreover, despite their biomimetic architectural excellence, these systems inevitably constitute simplified approximations of the extraordinarily complex *in vivo* physiological milieu, potentially obscuring critical intercellular communication networks, systemic immunological responses, and multi-organ interaction paradigms that characterize authentic pathophysiological processes. The standardization of experimental protocols across heterogeneous research institutions remains profoundly problematic, introducing significant methodological variability that compromises reproducibility standards and inter-laboratory validation paradigms. Perhaps most critically, the translation of promising organ-on-chip discoveries to successful clinical therapeutic interventions confronts the formidable challenge of traversing the substantial chasm between controlled in vitro microenvironments and the unpredictable complexity of human pathophysiological landscapes, where genetic polymorphisms, comorbidity interactions, and individual phenotypic variability introduce confounding variables that cannot be adequately recapitulated within microfluidic architectural systems.

Transcending their mechanistic revelations, these pioneering technological platforms function as extraordinary molecular discovery engines—unveiling novel therapeutic targets at the gut-kidney interface while enabling high-throughput screening of innovative therapeutic candidates under physiologically authentic conditions. These platforms represent the convergence of bioengineering excellence and translational medicine, establishing unprecedented opportunities for therapeutic innovation.

### 3.6 The translational imperative: navigating clinical implementation complexities

Contemporary gut-kidney axis interventional strategies confront formidable implementation challenges across multiple therapeutic dimensions: probiotic and prebiotic therapeutic efficacy demonstrates substantial variability contingent upon host microbiota individuality and metabolic phenotypic diversity; LPD regimens necessitate sophisticated, real-time nutritional status surveillance to prevent iatrogenic sarcopenia and protein-energy malnutrition; long-term fecal microbiota transplantation safety profiles remain incompletely characterized, particularly regarding immunological tolerance and microbiome stability.

**Figure 2:**
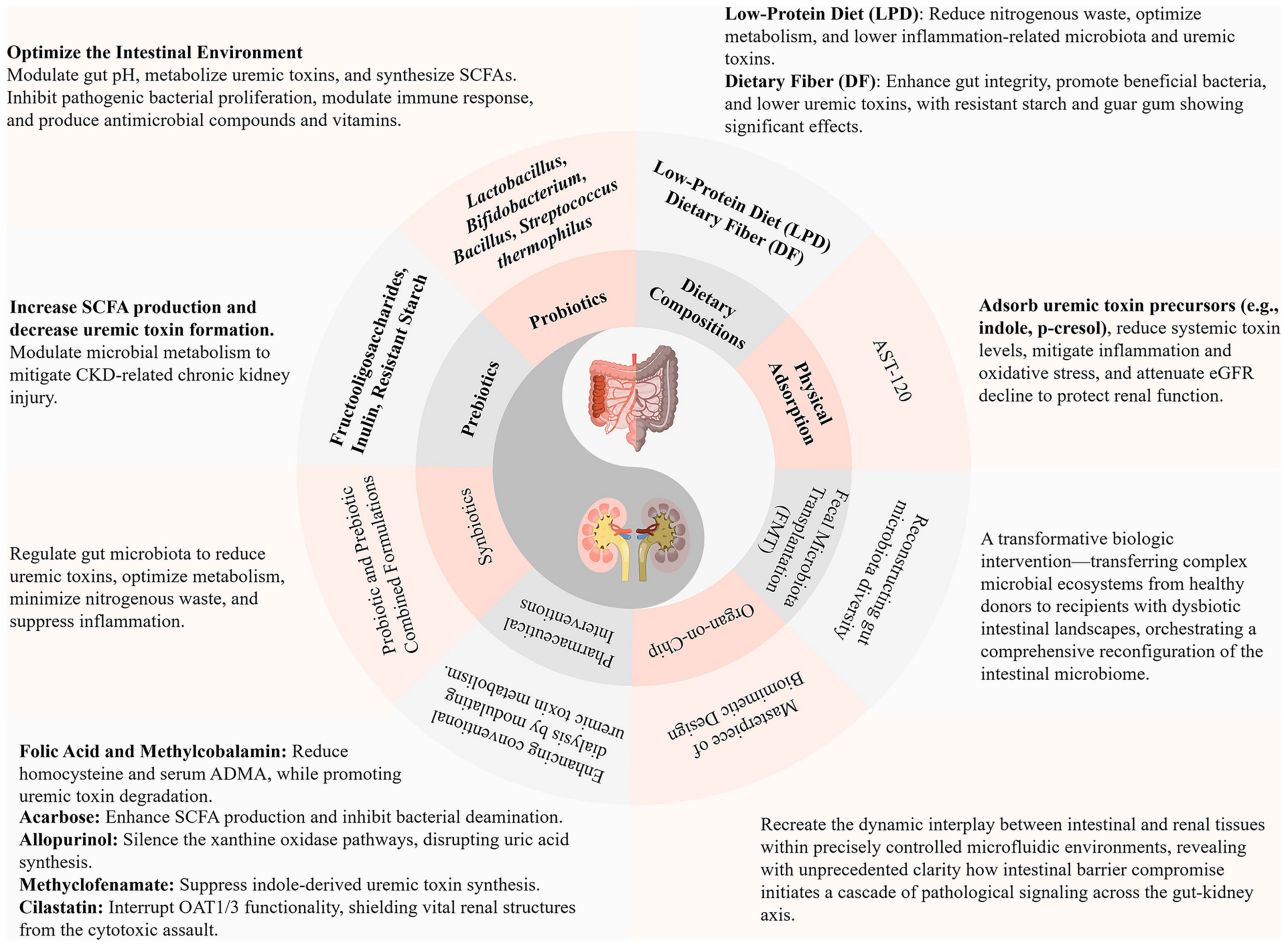
Therapeutic strategies targeting the gut-kidney axis: current advances. SCFAs, Short-Chain Fatty Acids; CKD, Chronic Kidney Disease; eGFR, Estimated Glomerular Filtration Rate; ADMA, Asymmetric Dimethylarginine; OAT1/3, Organic Anion Transporter 1/3.

Despite these translational complexities, these innovative therapeutic modalities represent a quantum leap in expanding the therapeutic armamentarium for chronic kidney disease management. The convergence of advancing precision medicine technologies, sophisticated microbiome characterization platforms, and personalized metabolic profiling heralds the emergence of individualized therapeutic protocols based on patient-specific gut microbiota signatures and host metabolic phenotypes as the paramount research imperative for future investigation (Figure 2).

This therapeutic evolution transcends conventional one-size-fits-all approaches, ushering in an era of precision gut-kidney axis medicine where therapeutic interventions are meticulously tailored to individual microbiome architectures and metabolic landscapes. The successful navigation of these translational challenges will fundamentally redefine chronic kidney disease management, transforming it from a reactive, symptom-driven discipline to a proactive, mechanism-based therapeutic science.

## 4 Therapeutic innovations and paradigmatic advances in traditional Chinese medicine: evidence-based insights through the gut-kidney axis framework

Contemporary evidence-based medical research has unequivocally established TCM as a therapeutic modality of unprecedented sophistication in CKD management—its integrative, precision-oriented, and holistic philosophical paradigm fundamentally transcends conventional therapeutic frameworks, effectuating profound amelioration of uremic toxin homeostasis and nephroprotective mechanisms (Li et al., 2019). This millennia-old medical tradition derives its conceptual edifice from the seminal classical compendium “Huangdi Neijing” (The Yellow Emperor’s Internal Canon), which articulates the foundational doctrine of “renal governance of congenital essence and splenic dominion over acquired essence,” demonstrating prescient recognition of the intricate visceral interconnectivity among spleen, kidney, and intestinal organ systems. The modern biomedical paradigm of the “gut-kidney axis” exhibits remarkable consonance with TCM’s integrative conceptual framework, elucidating the pathophysiological mechanisms underlying bidirectional renal-intestinal interactions in CKD pathogenesis. TCM therapeutic interventions provide multidimensional therapeutic strategies for comprehensive CKD management through sophisticated modulation of intestinal microbial ecosystems, fortification of intestinal mucosal barrier integrity, and nuanced regulation of inflammatory cascades. Spanning from meticulously isolated singular bioactive phytochemicals to synergistically formulated polyherbal compounds, Chinese medicinal preparations demonstrate extraordinary polypharmacological therapeutic capabilities characterized by multi-target engagement in CKD therapeutics (Ji et al., 2020; Wang et al., 2020).

### 4.1 Elucidating the therapeutic pharmacodynamics of individual botanical medicines

Rhubarb (Rhei Radix et Rhizoma) constitutes one of the most comprehensively investigated phytotherapeutic agents in CKD management, manifesting therapeutic efficacy through dual mechanistic pathways: facilitation of metabolic waste elimination and cultivation of balanced intestinal microbial ecosystems. Cutting-edge pharmacological investigations have elucidated rhubarb’s remarkable capacity to accelerate uremic toxin and creatinine clearance, thereby effectively mitigating the deleterious consequences of nitrogenous retention characteristic of renal insufficiency (Zeng et al., 2021). Of particular clinical significance is rhubarb’s profound modulatory influence on intestinal microbiome composition, demonstrating selective reduction of pathogenic bacterial genera including *Desulfovibrio*, *Clostridium*, and *Ruminococcus*, concurrent with enhancement of beneficial microbial populations such as *Parabacteroides*. Through inhibition of dysbiotic microbial proliferation, this intervention restores intestinal ecological equilibrium, attenuating aberrant protein fermentation processes and amino acid metabolic dysregulation (Wang et al., 2022). This sophisticated microbial homeostatic recalibration not only reinforces colonic barrier architectural integrity but also initiates cascading protective mechanisms, encompassing substantial reduction of uremic toxins. Consequently, rhubarb exerts pleiotropic therapeutic effects—simultaneously decelerating glomerulosclerotic progression and preventing renal interstitial fibrogenesis—thereby providing comprehensive therapeutic modalities for nephroprotection and functional preservation. Within the intricate gut-kidney axis pathological framework, these therapeutic mechanisms collectively demonstrate rhubarb’s multifaceted intervention across four critical junctures: orchestrating intestinal microbiome remodeling through selective bacterial modulation, reinforcing intestinal barrier integrity via colonic epithelial strengthening, optimizing microbial metabolite profiles through uremic toxin reduction and correction of aberrant fermentation processes, and ultimately providing direct renal cellular protection by attenuating both glomerulosclerotic progression and interstitial fibrogenesis.

Analogous to rhubarb’s regulatory mechanisms, Poria cocos exhibits distinctive targeted modulatory characteristics in optimizing the renal microenvironment. Experimental evidence substantiates Poria’s selective capacity to diminish bacterial populations intimately associated with proteinuric metabolism, specifically *Blautia*, *Escherichia-Shigella*, and *Bacteroides* (Guo et al., 2025). This precision-targeted regulation not only attenuates intestinal uremic toxin biosynthesis but also ameliorates renal metabolic burden through preservation of intestinal mucosal tight junction protein architecture. By alleviating metabolic burden, Poria facilitates renal stress mitigation, potentiating synergistic therapeutic effects, optimizing protein-energy utilization efficiency, and maintaining homeostatic fluid balance. The therapeutic trajectory of Poria cocos thus strategically targets three interconnected nodes within the gut-kidney pathological network: implementing selective microbiome modulation through specific reduction of proteinuria-associated bacterial populations, preserving intestinal barrier function via maintenance of tight junction protein architecture, and optimizing metabolite homeostasis through attenuation of intestinal uremic toxin biosynthesis, ultimately culminating in comprehensive renal stress alleviation and metabolic burden reduction.

Moreover, Danshen (*Salvia miltiorrhiza*) demonstrates exceptional bidirectional regulatory properties in the modulation of intestinal microbiome homeostasis. This botanical agent exhibits remarkable proficiency in significantly suppressing potentially pathogenic bacteria including *Bifidobacterium*, *Turicibacter*, *Streptococcaceae*, and *Desulfovibrio*, while concurrently promoting proliferation of beneficial microbial consortia, encompassing *Lactobacillus*, *Clostridium*, and *Ruminococcaceae* (Han et al., 2021). This equilibrium-oriented regulatory capacity not only rectifies aberrant amino acid metabolic pathway activation but also establishes systemic metabolic network modulation foundations for ameliorating renal functional impairment through restoration of energetic homeostasis. Danshen and Eucommia have additionally demonstrated significant nephroprotective functionality in diabetic nephropathy-related experimental models, inhibiting TGF-*β*/Smad signaling cascades to attenuate renal fibrogenesis progression (Wang et al., 2023a). The comprehensive therapeutic architecture of Danshen thus orchestrates intervention across four pivotal stages of the gut-kidney axis continuum: executing bidirectional microbiome regulation through simultaneous suppression of pathogenic bacteria and promotion of beneficial microbial consortia, correcting metabolic pathway dysfunction via rectification of aberrant amino acid metabolic networks, modulating inflammatory signaling cascades through targeted inhibition of TGF-β/Smad pathways, and providing direct renal cellular protection by attenuating fibrogenesis progression while restoring systemic metabolic homeostasis.

### 4.2 Pharmacochemical characterization of bioactive phytoconstituents

In contradistinction to crude botanical preparations, concentrated herbal extracts manifest substantially enhanced therapeutic efficacy, particularly in targeting specific pathophysiological mechanisms. Exemplifying this phenomenon, rhubarb’s anti-fibrotic properties are primarily attributable to its diverse anthraquinone constituents, encompassing emodin, rhein, aloe-emodin, chrysophanol, and physcion. These bioactive compounds synergistically exert anti-fibrotic, anti-inflammatory, antioxidant, and anti-apoptotic effects through multiple intracellular signaling cascades (Bi et al., 2025). Emodin demonstrates particularly pronounced efficacy in renal interstitial fibrosis inhibition (Xu L. et al., 2021; Feng et al., 2024), suppressing fibrogenetic progression through Klotho activation, SIRT3/FOXO3a pathway modulation, microbiota-endotoxin axis regulation, and epigenetic mechanisms including the lincRNA-ANRIL/let-7a/TGF-β1 network; rhein significantly inhibits TGF-β1/Smad2/3 signaling, blocking epithelial-mesenchymal transition in renal tubular epithelial cells while attenuating inflammatory mediator expression through NF-κB pathway suppression and enhancing autophagy via AMPK activation to ameliorate hyperglycemia-induced glomerulosclerosis; aloe-emodin regulates renal tubular cell apoptosis and proliferation through PI3K/Akt/mTOR pathway modulation, alleviating oxidative stress and interstitial fibrogenesis while conferring cytoprotection through mitochondrial functional stabilization; chrysophanol primarily activates the Keap1/Nrf2/HO-1 pathway to augment intracellular antioxidant capacity, diminish reactive oxygen species generation, and suppress TGF-β1 and *α*-SMA expression; physcion predominantly exerts anti-inflammatory and anti-fibrotic effects through STAT3 signaling pathway inhibition while concurrently modulating TLR4/MyD88/NF-κB cascades, demonstrating superior immunomodulatory efficacy in IgA nephropathy and nephrotoxic injury models (Feng et al., 2025).

Concerning the aforementioned Poria, its cortical tissues contain abundant tetracyclic triterpenoid compounds, particularly Pachymic acid and Poricoic acids, which confer significant nephroprotective effects against acute and chronic kidney injury through modulation of pivotal signaling networks including TGF-β1/Smad, Wnt/β-catenin, NF-κB, Keap1/Nrf2, and AQP2/ENaC pathways, effectively suppressing inflammatory responses and fibrogenesis while ameliorating acute or chronic renal functional and histopathological alterations (Guo et al., 2025).

Curcumin exemplifies this pharmacological paradigm with extraordinary precision and clarity. Curcumin administration orchestrates intestinal microbiome compositional remodeling through selective *Prevotellaceae* suppression while promoting *Bacteroidaceae* and *Ruminococcaceae* proliferation (Shen et al., 2017). Additionally, this polyphenolic compound combats opportunistic pathogen overgrowth including *Bacteroides* and *Escherichia-Shigella* while facilitating expansion of beneficial SCFA -synthesizing bacteria such as *Lactobacillus* (Xu X. et al., 2021). Through inhibition of excessive lipopolysaccharide and inflammatory mediator production, curcumin fortifies intestinal barrier integrity, attenuates uremic toxin translocation, and mitigates renal fibrogenesis and NLRP3 inflammasome activation (Xu X. et al., 2021).

Panax notoginseng saponins represent another therapeutically significant class of botanical extracts, conferring profound nephroprotection through sophisticated microbial community modulation. Investigations demonstrate these triterpene saponins reduce phylogenetic abundance across multiple bacterial phyla including *Firmicutes*, *Bacteroidetes*, *Proteobacteria*, and *TM7*, while substantially promoting beneficial bacterial species proliferation encompassing *Bacteroides*, *Lactobacillus*, *Butyrivibrio*, and *Eubacterium*. Concurrently, they attenuate pathogenic bacterial populations including *Escherichia-Shigella* and *Ruminococcaceae* (Mafra et al., 2021). This precision modulatory mechanism not only reestablishes microbial homeostasis but also plays instrumental roles in decelerating renal fibrogenesis progression in CKD patients through pro-inflammatory factor inhibition and intestinal barrier permeability protection (Mafra et al., 2021).

Hirudin demonstrates remarkable capacity for intestinal microbiome abundance modulation, encompassing *Lactobacillus reuteri*, *Escherichia coli*, *Ruminococcus callidus*, and *Clostridium celatum*, effectuating normalization of their ecological proportions. Furthermore, while preserving colonic tight junction protein expression, it inhibits NLRP3-ASC-caspase-1 inflammasome pathway activation in intestinal epithelial cells, thereby diminishing epithelial barrier compromise and uremic toxin translocation (Long et al., 2024).

Punicalagin (PU), a potent polyphenolic compound extracted from *Punica granatum*, exerts profound influences on intestinal microbiome architecture. This bioactive constituent significantly reduces *Proteobacteria* abundance while augmenting SCFA-producing bacterial proportions, including *Akkermansia*, *Lachnospiraceae*, and *Eubacterium_coprostanoligenes_group*. This ecological transformation results in elevated cecal SCFA concentrations, concomitant with reduced abundance of endotoxin-producing bacteria (*Desulfovibrionaceae*) and pathogenic species (*Ruminococcaceae_RC9_gut_group* and *Helicobacter*). Through comprehensive intestinal microbiome ecological restructuring, PU efficaciously reduces circulating lipopolysaccharide and diamine oxidase (DAO) concentrations, ameliorating inflammatory responses and alleviating metabolic dysregulation in DN models. Through these mechanisms, it reestablishes gut-kidney axis equilibrium, providing promising therapeutic strategies for managing renal dysfunction associated with metabolic pathologies (Hua et al., 2022).

Certain individual botanical extracts exhibit prebiotic characteristics. Exemplarily, polysaccharides derived from *Achyranthes bidentata* (ABPW1) function as prebiotics through selective *Bacteroides* abundance enhancement while diminishing *Rikenella*, *Alistipes*, *Laedolimicola*, and *Faecalibaculum* populations. This polysaccharide undergoes intestinal microbial degradation for SCFA biosynthesis, particularly facilitating propionate and isobutyrate production (Si et al., 2024). Another sulfated polysaccharide—fucoidan extracted from marine kelp—can reverse high-fat diet-induced intestinal microbiome diversity reduction. Following fucoidan supplementation, beneficial probiotic abundance including *Streptococcus*, *Lactobacillaceae*, and *Christensenellaceae* significantly increased. This was accompanied by substantial elevations in cecal SCFA concentrations, particularly acetate. SCFAs subsequently attenuate inflammation and oxidative stress through G protein-coupled receptor (GPR43) activation in renal tubular cells and podocytes, enhance mitochondrial functionality, and ameliorate renal complications including glomerular hyperfiltration and renal fibrogenesis (Zhong Z. et al., 2024).

Furthermore, in the extensively investigated CKD primary etiology of DN, herbal extracts demonstrate profound and comprehensive research foundations due to their phytochemical diversity and exceptional metabolic modulatory capabilities. Among polyphenolic compounds, Curcumins, Epigallocatechin-3-gallate, Resveratrol, and Chlorogenic acid; flavonoid constituents including Chrysin, Naringenin, and Total flavones of *Abelmoschus manihot*; quinone compounds exemplified by Tanshinone IIA; alkaloids typified by Berberine; and terpene compounds with Astragaloside IV demonstrating paramount efficacy—all achieve nephroprotection through endoplasmic reticulum stress pathway modulation. Lignan compounds such as Arctigenin and compound extracts including *Ginkgo biloba* extract EGB761 and total paeony glucosides similarly demonstrate therapeutic potential. These agents inhibit cellular apoptosis, augment antioxidant defense systems, attenuate inflammatory responses, and fundamentally ameliorate DN glucose metabolic dysregulation, thereby mitigating renal injury (Wei et al., 2023). Additional compounds including Acetylshikonin, *Abelmoschus esculentus*, Bupleurum polysaccharides, Notoginsenoside R1, *Coreopsis tinctoria*, Taxus chinensis, Dioscoreae Rhizoma, hedera saponin, and Acteoside have all demonstrated exceptional anti-fibrotic capabilities in experimental models (Wang et al., 2023a, 2025; Zhong X. et al., 2024).

This comprehensive phytoconstituent characterization unveils an extraordinarily sophisticated therapeutic paradigm wherein these meticulously orchestrated bioactive compounds execute precision-guided interventions across the quintessential sequential stages of gut-kidney axis pathophysiological cascades. At the primordial stage of intestinal microbiome dysbiosis, an elite cadre of compounds—encompassing curcumin, Panax notoginseng saponins, hirudin, and punicalagin—orchestrates exquisitely selective microbial community architectural remodeling, while specialized polysaccharide matrices, notably ABPW1 and fucoidan, manifest sophisticated prebiotic functionalities that catalyze the strategic proliferation of beneficial bacterial consortia. Progressing to the critical juncture of intestinal barrier dysfunction amelioration, this therapeutic triumvirate of curcumin, hirudin, and Panax notoginseng saponins functions as molecular guardians, meticulously preserving the structural sanctity of tight junction protein complexes and epithelial architectural integrity, thereby establishing formidable barriers against the pathological permeability escalation that epitomizes CKD pathogenesis. The subsequent phase of microbial metabolite dysregulation rectification involves polysaccharide-mediated facilitation of SCFA biosynthetic machinery—particularly optimizing propionate, acetate, and isobutyrate metabolic flux—while punicalagin concurrently executes precision targeting of circulating endotoxin neutralization and diamine oxidase level modulation. During the pivotal inflammatory cascade activation phase, a sophisticated arsenal of anthraquinone constituents, exemplified by rhein and physcion, implements strategic suppression of NF-κB and STAT3 signaling networks, while hirudin provides targeted inhibition of NLRP3 inflammasome activation, and triterpenoid compounds orchestrate comprehensive modulation of TLR4/MyD88 cascades, collectively establishing a coordinated attenuation of pro-inflammatory mediator expression throughout the entire gut-kidney axis continuum. Culminating in the resolution of renal cellular pathology, emodin, aloe-emodin, and chrysophanol deploy highly specialized mechanisms targeting renal fibrogenesis through synchronized TGF-β1/Smad pathway inhibition, Keap1/Nrf2 antioxidant axis activation, and mitochondrial functional stabilization, while an expansive therapeutic armamentarium—incorporating tanshinone IIA, berberine, and astragaloside IV—delivers comprehensive nephroprotective efficacy through an intricate symphony of synergistic mechanisms encompassing cellular apoptosis suppression, antioxidant capacity amplification, and fundamental metabolic dysregulation resolution, ultimately establishing a paradigmatic framework for precision medicine in CKD management.

### 4.3 Synergistic pharmacodynamics of botanical pair combinations

Based upon the classical TCM “sovereign-minister-assistant-courier” compatibility doctrine, traditional herbal pairs in CKD therapeutics demonstrate significant synergistic pharmacological effects. The Astragalus-Danshen combination has been definitively proven to substantially reverse intestinal microbial dysbiosis, characterized by increased Firmicutes/Bacteroidetes ratios and enhanced abundance of butyrate-producing bacterial species including *Akkermansia* and *Lactobacillus*. The mechanistic foundation of this synergistic interaction involves tryptophan metabolism regulation and butyrate biosynthetic pathway modulation, not only ameliorating renal metabolic dysregulation but also playing pivotal roles in decelerating renal fibrogenesis progression (Han et al., 2021). Another therapeutically potent combination—Astragalus and Panax notoginseng—suppresses NF-κB signaling pathway activity through *Bifidobacterium*-mediated Mincle receptor modulation, resulting in diminished M1 macrophage proportions while promoting M2 polarization. This immunomodulatory process significantly attenuates both renal and intestinal inflammatory responses, emphasizing this combination’s dual therapeutic roles in managing inflammation and fibrogenesis throughout CKD progression (Rui-Zhi et al., 2020). The Astragalus-Cordyceps combination effectively reverses intestinal microenvironmental dysbiosis in DN, significantly improving renal functional parameters with superior therapeutic efficacy compared to Astragalus monotherapy (Li J. et al., 2024).

### 4.4 Pharmacological investigations of polyherbal formulations and proprietary medicines

Classical TCM polyherbal formulations, predicated upon “syndrome differentiation and treatment” principles, generate synergistic therapeutic outcomes through harmonious multi-constituent interactions. Formulation ratios and therapeutic strategies are meticulously designed with profound scientific foundations and mathematical precision, establishing widespread clinical recognition. Exemplarily, Fuzheng Huayu Jiangzhuo Tongluo Decoction represents a sophisticated nine-herb formulation that exerts specific intestinal microbiome modulatory effects through selective reduction of *Monosporaceae*, *Lactobacillus*, and *Eubacterium nodatum* abundance. Additionally, this polyherbal compound significantly diminishes circulating concentrations of renal injury-associated metabolites including citric acid and bile acids. Its mechanistic therapeutic foundation correlates intimately with inflammatory cascade modulation and renal tubulointerstitial fibrogenesis inhibition, providing comprehensive therapeutic pathways for nephroprotection (Chen et al., 2022). Shulifenxiao Formula demonstrates potential as an alternative therapeutic option for hormone- and immunosuppressant-resistant nephrotic syndrome in refractory idiopathic membranous nephropathy, effectively ameliorating renal histopathological conditions (Cui et al., 2021). Mahuang Fuzi and Shenzhuo Decoction, targeting primary membranous nephropathy (MN), facilitate renal microenvironmental optimization and immune regulatory enhancement, thereby attenuating proteinuria and stabilizing renal functional parameters (Zhang N. et al., 2024). Extra formulations including Xiexin Decoction, ErHuang Formula, Qi-dan-di-huang Decoction, Danggui-Shaoyao-San, Danggui Buxue Decoction, and Sanzi Guben Decoction have been empirically validated in experimental models to confer nephroprotective effects through multiple mechanisms encompassing inflammatory attenuation, inflammatory cell chemotaxis and homing amelioration, NF-κB pathway activation suppression, and endoplasmic reticulum stress and apoptotic inhibition, thereby decelerating renal functional deterioration and preventing progressive fibrogenetic histopathological alterations (Wang et al., 2023a; Wei et al., 2023).

Traditional Chinese patent medicines (TCPMs), conceptually rooted in classical polyherbal formulations and refined through contemporary pharmaceutical technologies, provide standardized therapeutic approaches for specific pathological conditions while seamlessly integrating with modern medical practice. Yishen Qingli Heluo Granules (YQHG), representing a flagship TCM preparation for CKD management, serves as an exemplary model. Investigations demonstrate that YQHG treatment significantly enhances relative abundance of SCFA-producing bacterial species including *Lactobacillaceae*, *Lactobacillus*, and *Lactobacillus gasseri* in experimental CKD models. Furthermore, it elevates total intestinal SCFA concentrations (particularly acetate and butyrate), fortifying intestinal barrier integrity through permeability reduction and microbial translocation limitation. Microbiome transplantation studies further elucidate that YQHG’s nephroprotective mechanisms are partially mediated through intestinal microbiome modulation, specifically through bacterial populations involved in SCFA biosynthesis (Sun et al., 2022).

Hibiscus Capsules primarily comprise *Hibiscus mutabilis* floral extracts, abundant in diverse flavonoid compounds (Cai et al., 2017). These bioactive constituents undergo *in vivo* biotransformation to glucuronide-sulfate conjugates, possessing multifaceted pharmacological properties including metabolic enhancement, antihypertensive effects, antioxidative stress amelioration, and anti-inflammatory activities, conferring crucial nephroprotective roles in DN, hypertensive nephropathy, glomerulonephritis, and LN (Chen et al., 2016; Cao et al., 2022; Vagopoulou et al., 2024). Hibiscus Capsules demonstrate remarkable antiproteinuric efficacy and have received approval from the China National Medical Products Administration for chronic nephritis treatment (Li et al., 2021). Multicenter randomized controlled trials substantiate their profound antiproteinuric effects in patients with early-stage primary glomerulonephritis (Zhang et al., 2014) and IgA nephropathy (Li et al., 2020). In adenine-induced chronic renal failure experimental models, Hibiscus Capsules effectively prevent renal tubulointerstitial fibrogenesis through NADPH/ROS/Erk signaling pathway inhibition (Cai et al., 2017). Moreover, in 5/6 nephrectomy experimental models, they alleviate renal burden through tryptophan transport disruption and microbial metabolic modulation, thereby diminishing uremic toxin accumulation (Lu et al., 2021).

Yishen Huashi Granules (YSHS) demonstrate comparably impressive nephroprotective characteristics. Research reveals that YSHS treatment significantly reduces 24-h proteinuria in non-dialysis CKD patients while promoting beneficial intestinal microbial growth (e.g., *Faecalibacterium*, *Lachnospiraceae*, *Lachnoclostridium*, *Sutterella*) and inhibiting potentially pathogenic bacterial species (e.g., *Eggerthella*, *Clostridium innocuum*). Subsequent analysis demonstrates that *Lachnoclostridium* and *Lachnospiraceae* increases negatively correlate with 24-h proteinuria reduction. Additionally, YSHS intervention modulates multiple metabolic pathways encompassing N-glycan synthesis, steroid hormone biosynthesis, and biotin metabolism. Notably, microbial alterations associated with steroid hormone synthesis regulation exhibit gender-specific differences, with male patients demonstrating increased *Lachnoclostridium* and *Lachnospiraceae*, while female patients experience significant *Prevotella* elevations (Dong et al., 2024).

Additional commonly utilized TCM patent medicine formulations including Sanzi Guben Polysaccharides (SZPs), Fufang Zhenzhu Tiaozhi Capsules (FTZ) & FTZ Polysaccharides (FTZPs), QiDiTangShen granules (QDTS), Moshen granule (MSG), Qiwei granule, Tongxinluo, Liuwei Dihuang Pill, Chaihuang-Yishen granule, The Tangshen Formula, and Huangkui Capsule demonstrate capacity to attenuate renal fibrogenesis and histostructural damage in pathological conditions of CKD primary etiologies through modulation of diverse mechanistic pathways (Wang et al., 2023a, 2025; Li J. et al., 2024; Miao et al., 2024b, 2025).

TCM confers substantial therapeutic advantages in CKD management, primarily attributable to its polypharmacological capacity to engage multiple molecular targets and signaling pathways, functioning across diverse pathophysiological levels throughout disease progression. Whether through botanical decoctions, sophisticated formulations, or standardized patent medicines, TCM demonstrates therapeutic versatility through intestinal microbiome modulation, intestinal barrier integrity enhancement, and renal metabolic optimization. These multifaceted therapeutic effects precisely target pathophysiological mechanisms, providing efficacious and personalized treatment modalities. The pharmacoeconomic advantages and relatively minimal adverse reaction profiles associated with TCM are largely attributable to their compositional complexity and multi-target synergistic mechanisms. Chinese botanical medicines contain abundant bioactive compounds including phenolics, saponins, glycosides, flavonoids, alkaloids, tannins, steroids, and terpenes, each exhibiting distinct physiological effects. Exemplarily, botanical agents including rhubarb, Danshen, and turmeric demonstrate exceptional phenolic compound concentrations, exhibiting potent antioxidant and anti-inflammatory properties that underscore their tremendous potential in nephropathic management. In renal disease pathophysiology, oxidative stress and inflammatory responses play pivotal roles in mediating glomerular and tubular damage, ultimately precipitating renal functional decline. Excessive reactive oxygen species accumulation and antioxidant enzyme inhibition (particularly superoxide dismutase) can activate crucial signaling cascades including NF-κB and MAPK, subsequently promoting pro-inflammatory mediator synthesis and release (Tarek et al., 2023; Chu et al., 2024). Phenolic compounds in Chinese botanical medicines can ameliorate these pathological effects through ROS production inhibition and inflammatory signaling cascade suppression, thereby conferring therapeutic benefits in nephropathic conditions (Sahakyan et al., 2022). Additionally, Astragalus contains abundant saponins and glycosides, with specific astragalosides exhibiting potent anti-inflammatory and anti-fibrotic properties, while flavonoid constituents possess significant antimicrobial characteristics (Cui et al., 2023; Su et al., 2023; Ning et al., 2024). In AKI and CKD contexts, these compounds promote nephroprotection through diuretic, natriuretic, and direct renoprotective mechanisms, substantially improving renal functional parameters.

### 4.5 Acupuncture and moxibustion: non-pharmacological therapeutic modalities for CKD

Acupuncture and moxibustion, constituting non-pharmacological therapeutic modalities within TCM, represent integral components of Complementary and Alternative Medicine (CAM) in comprehensive CKD management. Acupuncture’s effectiveness has been rigorously validated through randomized controlled trials (Yu et al., 2017), while moxibustion’s therapeutic benefits are substantiated by numerous meta-analytical investigations (Lu et al., 2021). In CKD patients, acupuncture stimulation of specific acupoints (e.g., Hegu, Zusanli, Taixi) enhances renal hemodynamics and ameliorates renal functional parameters, encompassing eGFR elevation and serum creatinine reduction (Yu et al., 2017). Acupuncture additionally exerts profound modulatory influences on inflammatory biomarkers and immunological responses, attenuating intrarenal inflammation and immune-mediated injury. Through neuroendocrine system modulation, acupuncture alleviates oxidative stress, thereby addressing CKD-associated complications including hypertension, proteinuria, and fatigue, ultimately enhancing patient quality of life (Xiong et al., 2018).

Moxibustion involves combustion of *Artemisia vulgaris* (moxa) upon acupuncture needles, utilizing thermal energy, photonic radiation, and aromatic constituents’ therapeutic properties to generate beneficial physiological effects. Investigations demonstrate moxibustion’s capacity to improve local renal microcirculation and confer podocyte cytoprotection (Lu et al., 2021). Meta-analytical evidence demonstrates moxibustion significantly ameliorates serum creatinine concentrations, reduces urinary protein excretion, diminishes BUN levels, and enhances overall quality of life in CKD patients (Li et al., 2017; Zhou et al., 2020). Although effects on eGFR, creatinine clearance, and hemoglobin concentrations remain limited, moxibustion’s circulatory enhancement and inflammatory modulation capabilities establish it as a valuable adjunctive therapeutic modality for managing renal functional decline and alleviating CKD-associated symptomatology (Zhou et al., 2020).

### 4.6 Integrative therapeutic paradigms in contemporary CKD management

TCM provides comprehensive and holistic therapeutic paradigms for CKD through synergistic integration of pharmacological and non-pharmacological interventions. Whether through botanical medicines that modulate gut-kidney axis functionality and restore intestinal barrier integrity, or through acupuncture and moxibustion that attenuate inflammatory responses and enhance circulation, TCM demonstrates remarkable therapeutic efficacy in mitigating nephropathic pathological progression. TCM’s patient-centric approach transcends conventional disease management, emphasizing holistic health optimization and quality of life enhancement, thereby providing comprehensive supportive care systems for CKD patients.

However, the clinical implementation of TCM therapeutics in CKD populations necessitates extraordinary vigilance regarding safety profiles, pharmacokinetic alterations, and potential therapeutic interactions, particularly given the compromised renal clearance capacity and complex polypharmacy regimens characteristic of this patient demographic. CKD patients exhibit profound alterations in drug metabolism and elimination pathways, with significant reductions in renal organic anion transporter (OAT1/OAT3) and organic cation transporter (OCT2) functionality, potentially precipitating unexpected bioaccumulation of herbal constituents and their metabolites. The nephrotoxic potential of certain botanical compounds presents particular concern: aristolochic acid-containing herbs have been definitively associated with progressive tubulointerstitial nephritis and urothelial carcinogenesis, necessitating absolute contraindication in renal disease contexts (Dickman et al., 2023). Similarly, rhubarb’s oxalate content may exacerbate hyperoxaluria and calcium oxalate nephrolithiasis in susceptible individuals, while prolonged usage can precipitate electrolyte imbalances and melanosis coli (Kumar et al., 2021).

Herb-drug interactions constitute a critical consideration in CKD therapeutic paradigms, where patients typically require multiple concurrent medications including ACE inhibitors, ARBs, diuretics, phosphate binders, and immunosuppressive agents. Danshen demonstrates significant anticoagulant properties that may potentiate warfarin effects, increasing hemorrhagic risks (Zhang Y. et al., 2020), while Astragalus may enhance hypoglycemic medication effects, necessitating careful glucose monitoring in DN patients. Panax notoginseng exhibits antiplatelet aggregation properties that contraindicate concurrent usage with anticoagulant therapies (Zhao C. et al., 2025), while certain TCM formulations containing licorice may precipitate pseudohyperaldosteronism, exacerbating hypertension and fluid retention in volume-sensitive CKD patients (Sabbadin et al., 2024). The hepatic cytochrome P450 system, crucial for herbal constituent metabolism, demonstrates altered functionality in advanced CKD, potentially compromising the biotransformation of complex phytochemical matrices and increasing susceptibility to hepatotoxic reactions (Chen et al., 2021). Polyherbal formulations present additional complexity, as synergistic interactions among multiple bioactive constituents may unpredictably modulate pharmacokinetic profiles and therapeutic windows. Quality control concerns regarding standardization, adulteration with undisclosed synthetic compounds, and contamination with heavy metals or microbial pathogens represent persistent challenges in herbal medicine implementation, particularly problematic in CKD populations with compromised immune function and reduced toxin clearance capacity. Individualized therapeutic approaches must incorporate comprehensive assessment of residual renal function, concurrent medication regimens, and patient-specific risk factors including age, comorbidity burden, and genetic polymorphisms affecting drug metabolism. Regular monitoring of renal function parameters, electrolyte balance, liver enzymes, and complete blood counts becomes imperative during TCM intervention, with dose adjustments or discontinuation protocols established based on clinical response and adverse event emergence. The integration of traditional diagnostic methodologies with contemporary biomarker assessment and pharmacogenomic profiling may optimize therapeutic outcomes while minimizing iatrogenic complications.

**Figure 3:**
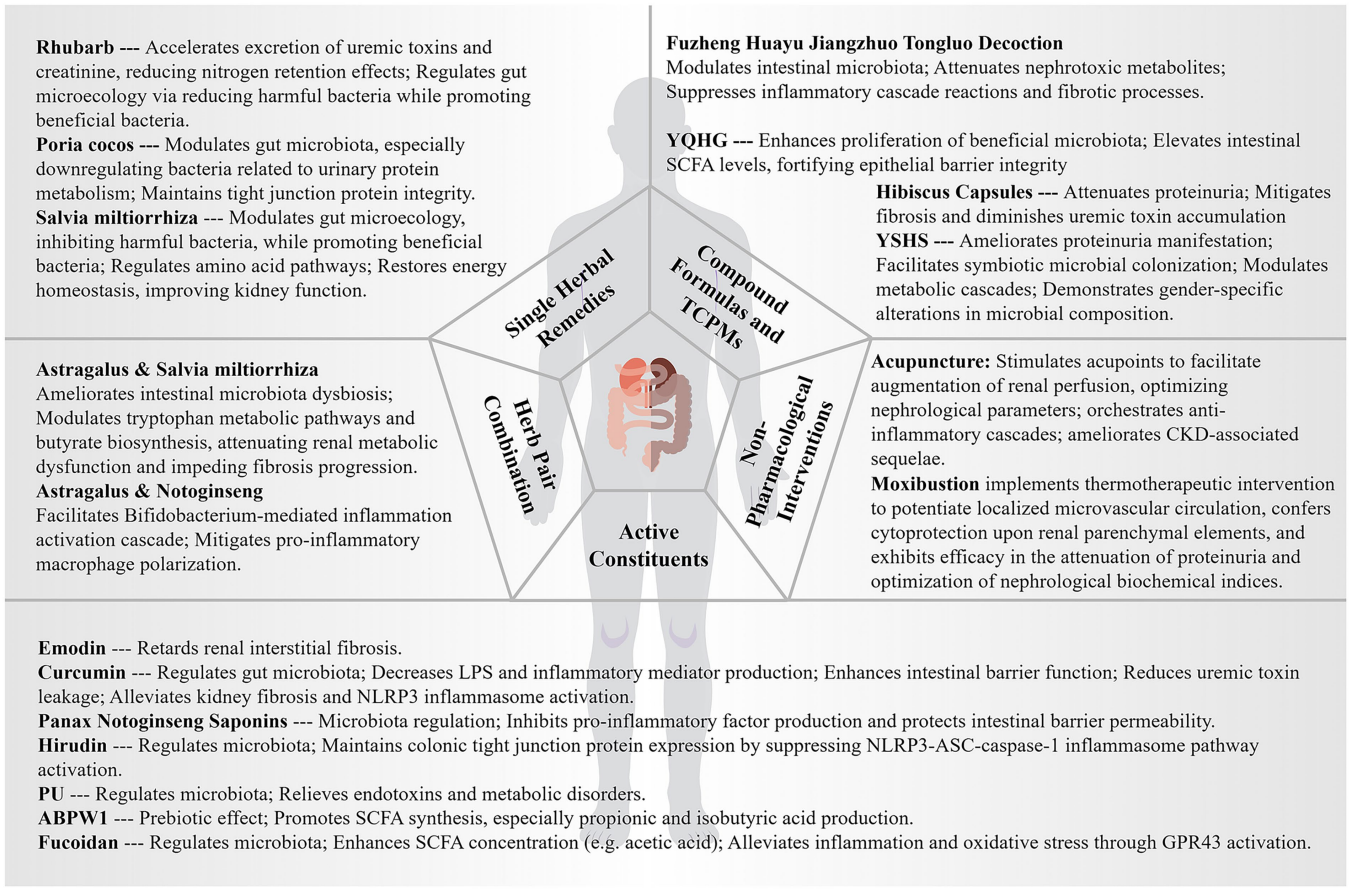
Progress and innovations in TCM: insights from the “Gut-Kidney Axis” theory. YQHG, *Yishen Qingli Heluo Granule*; YSHS, *Yi-Shen-Hua-Shi Granule*; CKD, Chronic Kidney Disease; LPS, Lipopolysaccharide; NLRP3, NOD-like receptor family pyrin domain containing 3; ASC, Apoptosis-associated speck-like protein containing a CARD; PU, Punicalagin; ABPW1, *Achyranthes bidentata* polysaccharide; SCFAs, Short-Chain Fatty Acids; GPR43, G protein-coupled receptors.

Professional supervision by qualified TCM practitioners with specialized nephrology expertise, in close collaboration with conventional nephrologists, represents the optimal paradigm for safe and effective herbal medicine implementation in CKD management. Patient education regarding potential adverse effects, drug interaction recognition, and the importance of full therapeutic disclosure to all healthcare providers constitutes an essential component of responsible integrative care. This multidimensional therapeutic approach, founded upon integrative philosophical principles while acknowledging inherent safety considerations and the imperative for rigorous clinical monitoring, represents a promising yet cautiously implemented paradigmatic advancement for future CKD therapeutic strategies, wherein the pursuit of therapeutic efficacy must be meticulously balanced against patient safety and the fundamental principle of “primum non nocere” (Figure 3).

## 5 Conclusion

The explication of the gut-kidney axis constitutes a transformative paradigmatic shift in our conceptualization of CKD pathophysiology, transcending conventional reductionist frameworks to embrace a more nuanced understanding of inter-organ metabolic orchestration. The metagenomic perturbations characteristic of CKD—manifesting as attenuated microbial biodiversity, compromised fermentative functionality, and deteriorated epithelial barrier architecture—precipitate a deleterious cascade whereby intestinal dysbiosis perpetuates and amplifies the uremic milieu through enhanced nephrotoxin biosynthesis and systemic pro-inflammatory signaling.

Traditional Chinese Medicine, distinguished by its intrinsic systems-based epistemological foundation and polypharmacological therapeutic sophistication, presents a uniquely advantageous pharmacological framework for modulating gut-kidney axis dysfunction. The phytochemical virtuosity inherent in TCM manifests through strategic reconstitution of intestinal microbial ecosystems, reinforcement of mucosal barrier homeostasis, and selective inhibition of uremic metabolite biosynthetic cascades. Archetypal botanical agents and their constituent bioactive principles—notably Rhubarb, Poria cocos, and *Salvia miltiorrhiza*—exhibit remarkable regulatory precision in orchestrating microbial community restructuring and metabolic equilibrium restoration. Sophisticated polyherbal formulations, exemplified by Yishen Qingli Heluo Granule and Yi-Shen-Hua-Shi Granule, actualize multidimensional therapeutic intervention through synergistic phytochemical architectures that harmonize intestinal-renal metabolic dialogue.

The contemporary convergence of cutting-edge computational intelligence and sophisticated bioengineering methodologies heralds transformative prospects for advancing TCM investigation within the gut-kidney axis paradigmatic framework. Artificial intelligence architectures facilitate extraordinarily nuanced deconvolution of the intricate tripartite relationships encompassing microbiome dynamics, metabolomic signatures, and therapeutic responsiveness, while intestinal organoid biotechnology furnishes physiologically authentic, high-throughput screening matrices for phytochemical evaluation, synergistically enabling the architectural construction of predictive algorithmic models for precision medicine implementation. Nevertheless, several formidable challenges persist in translating this paradigmatic understanding into clinically validated therapeutic interventions. The inherent heterogeneity of TCM formulations presents significant obstacles to standardization and reproducibility, while the intricate dose–response relationships governing phytochemical-microbiome interactions remain incompletely elucidated. Furthermore, the considerable inter-individual variability in baseline microbiome compositions and metabolic phenotypes complicates the development of universally applicable therapeutic protocols, necessitating more nuanced personalization strategies. Future investigative endeavors must prioritize the development of multi-omics integration platforms that can simultaneously capture microbiome taxonomic profiling, metabolomic fingerprinting, and pharmacokinetic-pharmacodynamic modeling to decipher the complex mechanistic networks underlying TCM efficacy. The emergence of precision fermentation technologies and synthetic biology approaches offers unprecedented opportunities for producing standardized bioactive compounds while preserving their therapeutic synergies. Additionally, the implementation of adaptive clinical trial designs, coupled with real-time microbiome monitoring and machine learning-guided dose optimization, promises to revolutionize the clinical validation of gut-kidney axis-targeted interventions.

This integrative therapeutic paradigm, synthesizing millennia of empirical pharmacological wisdom with contemporary microbiome genomics and cutting-edge technological innovations, illuminates a revolutionary approach to CKD therapeutics—one that targets fundamental pathophysiological mechanisms rather than superficial symptomatic amelioration through precision-guided intervention strategies. The gut-kidney axis thereby furnishes both mechanistic validation of TCM’s theoretical constructs and elucidates the molecular basis underlying its documented clinical efficacy, while advanced technologies provide the analytical framework necessary for translating traditional wisdom into contemporary precision medicine applications. These technological convergences, when synergistically integrated with traditional pharmacological wisdom, herald a new epoch in nephrology therapeutics wherein personalized, mechanistically-informed treatment paradigms will supplant conventional one-size-fits-all approaches, ultimately transforming CKD management through precision-guided restoration of gut-kidney metabolic homeostasis, establishing a robust foundation for the continued convergence of traditional and contemporary therapeutic modalities in nephrology practice.

## Glossary

ABPW1: *Achyranthes bidentata* polysaccharide
ACE: Angiotensin-Converting Enzyme
ADMA: Asymmetric dimethylarginine
AhR: Aryl hydrocarbon receptor
AKI: Acute Kidney Injury
Akt: Protein Kinase B
AQP2: Aquaporin-2
ARE: Angiotensin II Receptor Blocker
ASC: Apoptosis-associated speck-like protein containing a CARD
BCRP: Breast cancer resistance protein
BUN: Blood urea nitrogen
CAM: Complementary and Alternative Medicine
CFU: Colony forming unit
CKD: Chronic Kidney Disease
Col1a1: Collagen type I alpha 1 chain
COX-2: Cyclooxygenase-2
CRP: C-reactive protein
CRS: Cardiorenal Syndrome
CYP1A1: Cytochrome P450 1A1
DAO: Diamine oxidase
DF: Dietary fiber
DN: Diabetic nephropathy
EAA: Essential amino acid
ECM: Extracellular matrix
eGFR: Estimated glomerular filtration rate
ENaC: Epithelial Sodium Channel
eNOS: Endothelial Nitric Oxide Synthase
ESRD/ESKD: End-stage renal disease/End-stage kidney disease
F/B: Firmicutes/Bacteroidetes
FMT: Fecal microbiota transplantation
FOXO3a: Forkhead Box O3a
FSGS: Focal Segmental Glomerulosclerosis
FTZ: Fufang Zhenzhu Tiaozhi Capsules
FTZP: FTZ Polysaccharides
GCA: Glycocholic acid
GFR: Glomerular filtration rate
GG: Guar Gum
GPR43: G protein-coupled receptor 43
HIA: Hippuric Acid
HO-1: Heme Oxygenase-1
IAA: Indole-3-acetic acid
IDO: Indoleamine 2,3-Dioxygenase
IgAN: IgA nephropathy
IgAV-N: IgA vasculitis with nephritis
IKK: IκB kinase
IL-1/6/10/17: Interleukin-1/6/10/17
IMN: Idiopathic Membranous Nephropathy
iNOS: Inducible Nitric Oxide Synthase
IRE1*α*: Inositol-requiring enzyme 1α
IS: Indoxyl sulfate
JNK: c-Jun N-terminal kinase
Keap1: Kelch-like ECH-associated Protein 1
LN: Lupus Nephritis
LPD: Low-protein diet
LPS: Lipopolysaccharide
MAPK: Mitogen-activated protein kinase
MCD: Minimal Change Disease
MN: Membranous Nephropathy
MRP2/4: Multidrug resistance-associated protein 2/4
MSG: Moshen Granule
MsPGN: Mesangial proliferative glomerulonephritis
mTOR: Mechanistic Target of Rapamycin
NADPH: Nicotinamide adenine dinucleotide phosphate
NF-κB: Nuclear factor kappa B
NH₃: Ammonia
NLRP3: NOD-like receptor family pyrin domain containing 3
Nrf2: Nuclear Factor Erythroid 2–Related Factor 2
NO: Nitric oxide
OAT1/3: Organic anion transporter 1/3
OCT2: Organic cation transporter
PAG: Phenylacetic acid
PAMPs: Pathogen-Associated Molecular Patterns
PCS/PS: p-Cresyl sulfate
PD: Peritoneal Dialysis
PERK: PKR-like ER kinase
PHGG: Partially hydrolyzed guar gum
PI3K: Phosphoinositide 3-Kinase
PU: Punicalagin
QDTS: QiDiTangShen Granules
RCT: Randomized controlled trial
ROS: Reactive oxygen species
RS: Resistant starch
SCFA: Short-chain fatty acid
SBA: Secondary bile acid
SIRT3: Sirtuin 3
SP: Sodium propionate
STAT3: Signal Transducer and Activator of Transcription 3
SZP: Sanzi Guben Polysaccharides
TCA: Tricarboxylic acid
TCDCA: Taurochenodeoxycholic acid
TCM: Traditional Chinese Medicine
TCPM: Traditional Chinese Patent Medicine
TDO: Tryptophan 2,3-Dioxygenase
TGF-β1: Transforming growth factor beta 1
TLR: Toll-like receptor
TMA: Trimethylamine
TMAO: Trimethylamine N-oxide
TNF-α: Tumor necrosis factor alpha
YQHG: Yishen Qingli Heluo Granule
YSHS: Yi-Shen-Hua-Shi Granule
ZO-1: Zonula occludens-1
